# Family nurture intervention in the NICU increases autonomic regulation in mothers and children at 4-5 years of age: Follow-up results from a randomized controlled trial

**DOI:** 10.1371/journal.pone.0236930

**Published:** 2020-08-04

**Authors:** Martha G. Welch, Joseph L. Barone, Stephen W. Porges, Amie A. Hane, Katie Y. Kwon, Robert J. Ludwig, Raymond I. Stark, Amanda L. Surman, Jacek Kolacz, Michael M. Myers

**Affiliations:** 1 Department of Pediatrics, Columbia University Medical Center, New York, NY, United States of America; 2 Department of Psychiatry, Columbia University Medical Center, New York, NY, United States of America; 3 Department of Psychiatry, University of North Carolina at Chapel Hill, Chapel Hill, NC, United States of America; 4 Traumatic Stress Research Consortium, Kinsey Institute, Indiana University, Bloomington, IN, United States of America; 5 Department of Psychology, Williams College, Williamstown, MA, United States of America; University of British Columbia, CANADA

## Abstract

**Background:**

Maturation of multiple neurobehavioral systems, including autonomic regulation, is altered by preterm birth. The purpose of this study was to determine the long-term effects of Family Nurture Intervention (FNI) in the NICU on autonomic regulation of preterm infants and their mothers.

**Method:**

A subset of infants and mothers (48% of infants, 51% of mothers) randomly assigned to either standard are (SC), or SC plus the FNI in the NICU in a prior RCT (ClincalTrials.gov; NCT01439269) returned for follow-up assessments when the children were 4 to 5 years corrected age (CA). ECGs were collected for 10 minutes in mothers and their children while children were in their mothers’ laps. Heart rate, standard deviation for heart rate, respiratory sinus arrhythmia (RSA)–an index of parasympathetic regulation, and a measure of vagal efficiency were quantified.

**Results:**

Both children and mothers in the FNI group had significantly greater levels of RSA compared to the SC group (child: mean difference = 0.60, 95% CI 0.17 to 1.03, p = 0.008; mother: mean difference = 0.64, 95% CI 0.07 to 1.21, p = 0.031). In addition, RSA increased more rapidly in FNI children between infancy and the 4 to 5-year follow-up time point (SC = +3.11±0.16 log_e_ msec^2,^ +3.67±0.19 log_e_ msec^2^ for FNI, p<0.05). These results show that the rate of increase in RSA from infancy to childhood is more rapid in FNI subjects.

**Conclusion:**

Although these preliminary follow-up results are based on approximately half of subjects originally enrolled in the RCT, they suggest that FNI-NICU led to healthier autonomic regulation in both mother and child, when measured during a brief face-to-face socioemotional interaction. A Pavlovian autonomic co-conditioning mechanism may underly these findings that can be exploited therapeutically.

## Introduction

Approximately 10% of infants are born before 36 weeks in the US [1]. Despite remarkable medical progress, these preterm infants are at risk for multiple adverse neurobehavioral outcomes [2, 3]. Paralleling these issues, preterm infants also have altered function of the autonomic nervous system (ANS). Compared with babies born at term age, preterm infants display less vagal regulation of heart rate (HR), as well as alterations in sympathetic modulation of blood pressure [4, 5]. As young adults, preterm infants have lower levels of beat-to-beat heart rate variability (HRV) and slower HR recovery from exercise, all potential markers of unhealthy autonomic function [6].

Maternal factors, epigenetics, and the intrauterine environment, as well as the early postnatal experience, all shape the developing central and autonomic nervous systems. Dysfunction of these systems is implicated in the subsequent development of neuropsychiatric disorders and poorer physical health throughout the lifespan [7, 8]. There is growing evidence that the physical and emotional separation of infants and mothers inherent to care in the Neonatal Intensive Care Unit (NICU) contributes to these long-term adverse autonomic outcomes [9–11]. As a consequence, some interventions and care practices involving mother/infant physical and emotional contact have been implemented in an effort to improve neurobehavioral outcomes in preterm infants.

One widely practiced NICU intervention is skin-to-skin contact (SSC) between mother and infant. A 2016 Cochrane review of early SSC in the hospital supported the use of early SSC between healthy mothers and infants to promote breastfeeding, but concluded that more rigorously designed studies with larger sample sizes were necessary [12]. However, the review did include two studies of preterm infants that reported infant autonomic regulation outcomes. Infants who received SSC immediately after birth were more likely to achieve cardiorespiratory stabilization in the first hours after birth [13, 14].

Few interventions involving mother/infant interactions in the NICU have rigorously tested autonomic function over time. In one study of preterm infants, it was found that SSC led to more rapid maturation of cardiac vagal tone between 32 and 37 weeks [15]. A follow-up assessment at 10 years of age found significantly increased amplitude of RSA and attenuated stress responses. In addition, mothers’ anxiety in the SSC group was reduced when their preterm infants were 6 to 10 years of age [16].

To address these issues, the Nurture Science Program at Columbia University Medical Center (nurturescienceprogram.org) conducted a randomized controlled trial (RCT) of another NICU-based intervention, Family Nurture Intervention (FNI) comparing standard care alone (SC) with SC plus FNI (ClinicalTrials.gov, NCT01439269). FNI-NICU is aimed at facilitating *emotional connection* between infants and mothers during their traumatic early separation in the NICU [17]. Specially trained Nurture Specialists, who in this study were former NICU nurses, initiated FNI, on average, 1 week after birth while the infant was still in the incubator. The central focus of FNI is to help mothers and their infants emotionally connect via repeated engagement in “calming sessions.” During these ~1 hour-long sessions, mothers were encouraged to express a full range of feelings and speak emotionally with their infants in their native language. Initially, these sessions involved making contact with their infants through the ports of the incubator, using firm and sustained touch. Later, these sessions occurred outside the incubator with the mother holding their infant while in a comfortable chair. Skin-to-skin holding was encouraged for these sessions but some mothers preferred clothed holding. In addition to calming sessions prior to leaving the unit, mothers were given two small cotton cloths, one to wear in her bra and the other to place under the head of her infant, which they were encouraged to exchange daily. On average, FNI mothers engaged in these activities for 6 hours/week [18].

While the intervention encourages some techniques often practiced in the NICU, such as SSC, FNI does not rely solely on SSC and can be initiated while the infants are still confined to the incubator. Indeed, the first deep emotional exchange between infant and mother often took place while the infant was confined to the incubator via comfort touch and maternal emotional expression.

We previously reported many significant results from the FNI-NICU trial [11]. FNI was found to be feasible and safe [19]. FNI mothers had increased maternal sensitivity during normal care-giving activities toward the end of the NICU stay [20], and decreased symptoms of maternal anxiety and depression at 4 months infant corrected age (CA) [21]. Near term, FNI infants showed significantly increased high frequency electroencephalogram (EEG) power [22], altered EEG-coherence [23], and advanced maturation of brain activity [24]. At 18 months of age, FNI infants had improved cognitive and language scores on the Bayley, fewer attention problems on the Child Behavioral Check List, and decreased risk for socioemotional problems as assessed by the Modified Checklist for Autism in Toddlers (MCHAT) [25].

Recently, we reported that FNI infants demonstrated more rapid increases in parasympathetic function from ~35 weeks to ~41 weeks postmenstrual age [26]. In this follow-up study of a the FNI-NICU cohort, we tested the hypothesis that this unique intervention for mothers and infants in the NICU would lead to greater levels of parasympathetic regulation at 4 to 5 years CA. Theoretical considerations and clinical implications are discussed.

## Method

### Subjects

**Fig 1:**
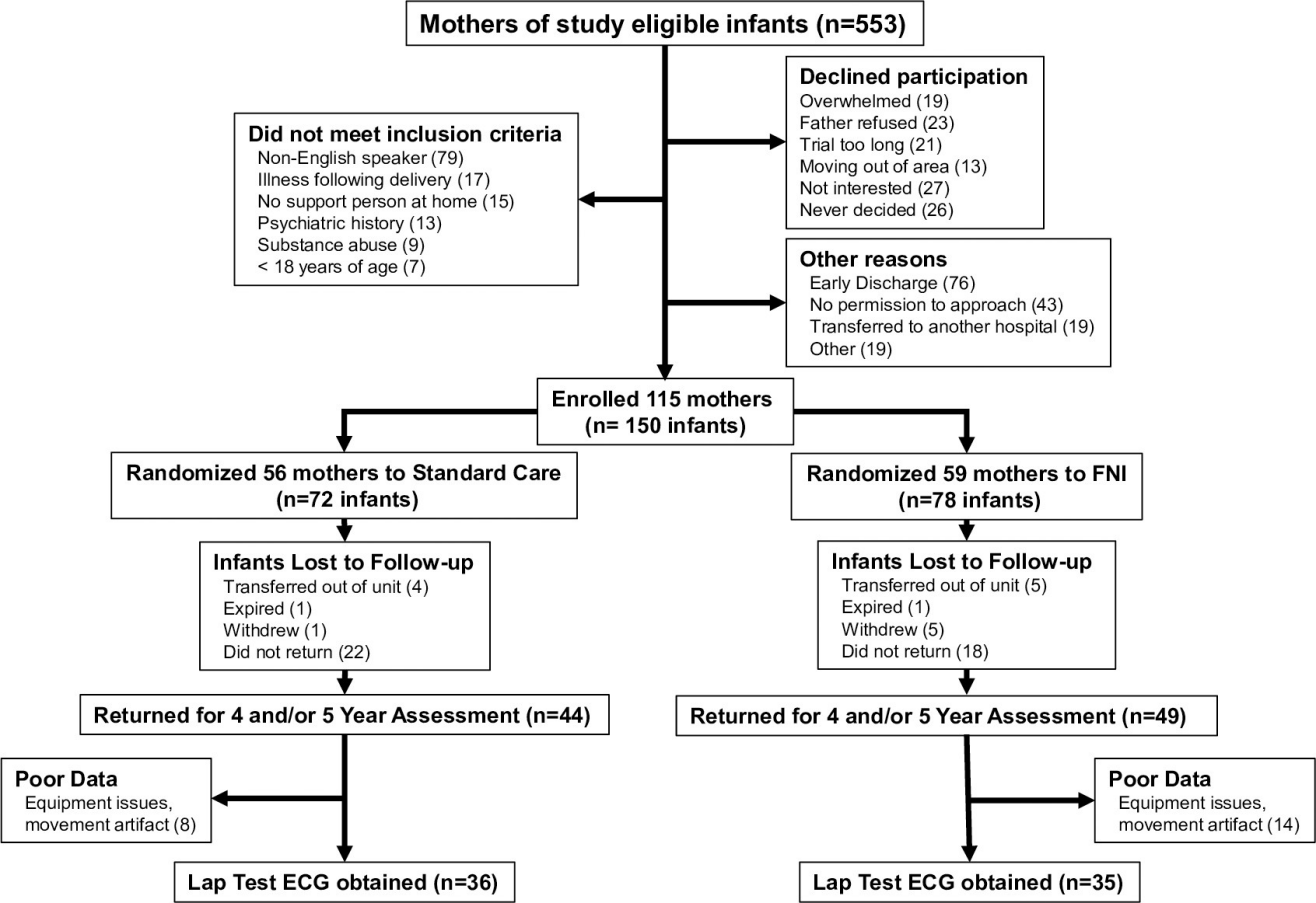
Consort flow diagram.

The data for these analyses were obtained from a subset of the 115 mothers and 150 infants originally recruited into a randomized controlled trial of the Family Nurture Intervention (FNI = 78) or standard NICU care (SC = 72) (Fig 1). This was a single center, parallel-group RCT conducted in the level IV NICU at Morgan Stanley Children’s Hospital of New York. Subjects for this trial were preterm infants born between 26 and 34 weeks of gestation. The trial enrolled 72 infants into the SC group and 78 infants into the intervention group [27].

Of the 56 mothers enrolled into the SC group, 40 gave birth to a single baby and 16 gave birth to twins. Thus, the 72 SC infants were comprised of 40 singletons and 32 were twins. Of the 59 mothers enrolled into the FNI group, 39 gave birth to a single baby and 20 gave birth to twins. For one mother in the FNI group who gave birth to twins, one of twins died prior to enrollment thus, for the FNI group, the 78 infants were comprised of 39 singletons and 39 twins.

The Columbia University Medical Center IRB approved all recruitment, consent, and study procedures on September 24, 2008. Mothers provided written informed consent for themselves and their infants. This study was initiated in January of 2009 with the last enrollment occurring in June of 2012. The 4–5 year follow-up assessments occurred during the period from December of 2013 through January of 2018.

On September 23, 2011 approximately half way through recruitment, the trial was registered (NCT01439269). At the time this trial began, we were unaware of the International Committee of Medical Journal Editor’s (ICMJE) position on this matter. Indeed, the position of the National Institutes of Health, which guided our IRB and Clinical Trials Office, was not clear at the time we initiated the trial. As soon as we became aware of the ICMJE’s position we registered the trial and did not alter the protocol after registration other than to expand the inclusion age range of the infants from 28–32 weeks gestational age (GA) to 26–34 weeks GA, which was reported as required. Thus, although trial registration occurred after initiation of enrollment, the protocol was not changed subsequent to the beginning of recruitment, and outcomes were not affected. The authors confirm that all ongoing and related trials for this intervention are registered.

### Family Nurture Intervention and standard NICU care

Study staff met with mothers assigned to the FNI group at the earliest time point after delivery to the intervention procedures. FNI aims to facilitate an emotional connection between mother and infant via calming sessions that include comfort touch, and communication of affect both in the incubator and during clothed contact and SSC. Mothers and infants in the SC group received medical and nursing care that is typical for our NICU; routine contact with medical staff, bedside nursing staff, and access to a psychologist and social worker. Additional study staff who were not responsible for facilitating the intervention met with mothers in both groups once weekly throughout NICU hospitalization to administer questionnaires. See [27] for a comprehensive report of the FNI protocol.

### 4–5 year assessments

Study subjects were contacted when children were within their 4^th^ and 5^th^ year CA and asked to return to the Nurture Science Laboratory for a follow-up visit. It was not possible to blind either the Nurture Specialists or mothers during the intervention phase of the research. However, the research staff involved in conducting the 4–5 year follow-ups were blinded to group assignment.

### ECG acquisition and lap test

Participants were connected to recording equipment shortly after arriving for the visit. Mothers and children were then brought into an empty room that contained only a large, comfortable chair. Mothers were asked to place their child on their lap facing one another. They were instructed to remain facing each other for 10 minutes and asked to “interact as they would normally.” During this period, child and mother ECGs were digitized at 1000 samples/sec using AcqKnowledge software and the MP150 base system with BioNomadix 2Ch Wireless ECG Amplifier from BIOPAC Systems, Inc. (Goleta, CA).

### Physiology processing

R-waves from the digitized ECG waveforms were marked using LabChart (ADInstruments, Dunedin, New Zealand). This was done in a 2-step process, first using Labchart’s automatic R-wave detection module, followed by visual inspection of the file to correct misplaced marks. From this software, an inter-beat-interval file was generated. These files were further edited to correct or remove segments of movement artifact using CardioEdit (Brain-Body Center, University of Illinois at Chicago, Chicago, IL). Finally, HR and HRV parameters were analyzed using CardioBatch (Brain-Body Center, University of Illinois at Chicago, Chicago, IL).

Parameters derived by CardioBatch from the corrected R-to-R intervals included HR (bpm), heart period (R-R intervals in msec), the standard deviation of HR (bpm) and respiratory sinus arrhythmia (RSA, in log_e_ msec^2^). The variance of HR occurring within the frequencies of spontaneous breathing was used as a metric for RSA. This procedure was based on the pioneering research in the electrophysiology of the vagus conducted by Hering [28]. Hering discovered that only the specific vagal fibers that were cardioinhibitory had a respiratory rhythm. Thus, quantifying the variance in HR within the frequencies of spontaneous breathing provides the functional measure of Hering’s observation. These metrics were computed both in 10 second epochs (for estimates of V_eff_) and in 30 second epochs. The average of the 30 epochs for each child was computed for each of the above parameters. Using the multiple values derived from the 10-second epochs, slopes of the relationship between heart period (i.e., epoch mean of the R-to-R intervals) and RSA for each child and mother were computed as a marker of the efficiency of vagal regulation of dynamic HR [29]. That is, a greater change in heart period per change in RSA, is taken as an index of greater efficiency of vagal modulation of HR.

### Data analyses

Group differences for all variables were determined using analyses of variance. Covariates included factors unrelated to the intervention that might have influenced the child’s and/or mother’s physiology: gestational age at birth, birth weight, age in months at the time of assessment (adjusted for weeks premature), sex, and twin status (Y/N) as covariates.

A second set of analyses were conducted to ensure that significant group differences were not biased due to inclusion of multiple children from a single mother (i.e. twins). Included in these analyses was data from singletons plus data from only one subject from each set of twins. Results presented in this article were not the primary outcome of the above described RCT, rather they were additional measures obtained 4 to 5 years after the primary outcome was obtained. Due to excessive movement artifact not all children provide usable data during this period. For children with more than one follow-up study we included results from the study that was closest to 60 months CA and that had usable ECG data. These numbers reflect inclusion of twins when both twins were assessed. Because twins may not represent independent samples and therefore inflate the degrees of freedom for the analyses, we repeated these analyses when only one twin was included.

## Results

Of the original 150 infants from 115 families, 93 children (44 SC, 49 FNI) and their mothers returned for a ~4-year, ~5-year, or both 4-year and 5-year follow-up studies. Data from 71 children (36 SC, 35 FNI) were included in these analyses. Among these were 29 twins (15 SC, 14 FNI). There were 11 sets of twins (6 SC, 5 FNI) for which data from both twins were available and 7 twins (3 SC, 4 FNI) for which data from only 1 twin was available. In one series of analyses of children, all 71 subjects were included.

For the second set of analyses excluding second twins, 30 SC and 30 FNI child values were included. There were 55 maternal assessments; however, 10 of these mothers were assessed twice, once with each twin. For the 11^th^ set of twins, both twins were assessed, but the mother’s data was usable for only one of these tests. Thus, in total 45 mothers were included in these analyses. As for children, two sets of analyses were performed using maternal data. The first included all 55 assessments with 10 mothers included twice, once with each twin. A second set of analyses included data recorded from 45 mothers; 31 mothers (15 SC, 16 FNI) while they were with their singleton child, 11 mothers while they were with twin A, and 3 mothers while with twin B.

The current analyses are based on ECG data obtained during a 10-minute “lap test” (see below). In total, data from 71 children (36 SC and 35 FNI) were included. ECGs of the mothers of these children were also recorded during the lap test. However, due to excessive movement and equipment failure, data from only 45 mothers (23 SC, 22 FNI) were usable. Note that some mothers with twins were assessed twice, once with each twin thus providing a total of 55 maternal recordings (29 SC, 26 FNI). Table 1 provides a summary of infant and maternal demographic characteristics for the subjects included in these analyses vs those who were not. These variables differed only with regard to income. Reported earnings were higher among included FNI families than among those excluded. Among SC families, those included reported a greater proportion of income at the two extremes, while for both study groups, families that were excluded were more likely to have opted not to disclose income information. Table 2 summarizes perinatal clinical characteristics of infants who did and did not return for the follow-up visit(s) at 4 to 5 years corrected age.

**Table 1:** Demographic variables of mothers, fathers and families who were included (n = 60) or not included in the current analyses (n = 55).

	Included		Excluded				
Family Characteristics	SC (N = 30)	FNI (N = 30)	SC (N = 26)	FNI (N = 29)	*F*		p
	Mean (SD)	Mean (SD)	Mean (SD)	Mean (SD)			
Mothers’ Age (years)	33.6 (5.4)	34.3 (6.1)	32.0 (5.9)	34.3 (6.2)	0.89		0.45
Fathers’ Age (years)	35.6 (6.2)	38.5 (9.1)	34.0 (6.7)	36.0 (7.0)	1.80		0.15
	N (%)	N (%)	N (%)	N (%)	*χ^2^*		*p*
Married	21 (70.0)	22 (73.3)	18 (69.2)	19 (65.5)	0.04		0.93
Mothers’ Ethnicity							
Black	6 (20.0)	5 (16.7)	7 (26.9)	8 (27.6)			
Hispanic	9 (30.0)	10 (33.3)	5 (19.2)	7 (24.1)	2.83		0.97
White	13 (43.3)	12 (40.0)	11 (42.3)	11 (37.9)			
Other	2 (6.7)	3 (10.0)	3 (11.5)	3 (10.3)			
Fathers’ Ethnicity							
Black	7 (23.3)	3 (10.0)	8 (30.8)	9 (31.0)			
Hispanic	7 (23.3)	12 (40.0)	5 (19.2)	3 (10.0)	11.17		0.26
White	12 (40.0)	13 (43.3)	9 (34.6)	14 (48.3)			
Other	4 (13.3)	2 (6.7)	4 (15.4)	3 (10.3)			
Mothers’ Education							
High School or lower	4 (13.3)	2 (6.7)	2 (8.0)	5 (17.9)			
Some College	5 (16.7)	7 (23.3)	9 (36.0)	5 (17.9)	5.17		0.52
Graduate or higher	21 (70.0)	21 (70.0)	14 (56.0)	18 (64.3)			
Fathers’ Education							
High School or lower	7 (23.3)	5 (16.7)	7 (28.0)	8 (28.6)			
Some College	5 (16.7)	8 (26.7)	5 (20.0)	3 (10.7)	3.39		0.76
Graduate or higher	18 (60.0)	17 (56.7)	13 (52.0)	17 (60.7)			
Family Income							
< $40,000	8 (26.7)	3 (10.0)	5 (19.2)	7 (24.1)			
$40,000 - $70,000	1 (3.3)	10 (33.3)	2 (7.7)	3 (10.0)	18.36		0.03
> $70,000	19 (63.3)	16 (53.3)	15 (57.7)	14 (48.3)			
Did Not Report	2 (6.7)	1 (3.3)	4 (15.4)	5 (17.2)			

Mean ± SD for mothers’ and fathers’ years is reported. Abbreviations: FNI, Family Nurture Intervention; SC, standard care. F and p values reflect results comparing means or proportions across all four groups by characteristic (e.g. Mothers’ Ethnicity df = 9).

**Table 2:** Perinatal clinical characteristics of infants who did (Included N = 71) and did not (n = 57) return for the follow-up visit(s) at 4 to 5 years corrected age, or who returned but did not provide usable data during the lap test (n = 22) (Excluded = 79).

		Included		Excluded			
Infant Characteristics		SC (N = 36)	FNI (N = 35)	SC (N = 36)	FNI (N = 43)		
		Mean ± SD	Mean ± SD	Mean ± SD	Mean ± SD	*F*	*p*
Gestational age (wk)		31.0 (2.4)	30.5 (2.1)	30.4 (2.7)	31.1 (2.2)	0.852	.468
Birth weight (gr)		1451 (404)	1412 (470)	1406 (413)	1449 (417)	0.764	.516
Length at birth (cm)		40.1 (4.2)	40.0 (3.2)	39.3 (3.8)	39.7 (3.6)	0.353	.787
Head Circumference (cm)		28.6 (3.07)	28.1 (2.7)	27.5 (3.0)	28.4 (2.5)	0.936	.425
		**N (%)**	**N (%)**	**N (%)**	**N (%)**	***χ^2^***_***df(3)***_	***p***
Male		18 (50.0)	18 (51.4)	18 (50.0)	23 (53.5)	0.131	.988
Singleton		21 (58.3)	20 (57.1)	19 (52.8)	19 (44.2)	1.986	.575
Cesarean Delivery		23 (63.9)	26 (74.3)	25 (69.4)	36 (83.7)	4.309	.230
Resuscitated at birth		10 (27.8)	11 (31.4)	8 (22.2)	10 (23.3)	1.031	.794
Placed on CPAP at birth		36 (100)	33 (94.3)	32 (88.9)	37 (88.1)	5.000*	.172
Apgar Scores	≥4 at 1 minute	32 (88.9)	30 (85.7)	35 (97.2)	41 (95.3)	4.490*	.213
	≥7 at 1 minute	29 (80.6)	24 (68.6)	29 (80.6)	32 (74.4)	1.937	.586
	≥4 at 5 minutes	36 (100)	35 (100)	36 (97.1)	43 (100)	2.933*	.347
	≥7 at 5 minutes	35 (97.2)	33 (94.3)	34 (94.4)	42 (97.7)	0.026*	.810

* *Likelihood Ratio*.

The F, χ^2^ and p values are from ANOVA and χ^2^ tests comparing the 4 (included vs. excluded) x (SC vs. FNI) infant categories. Abbreviations: CPAP, continuous positive airway pressure; LOS, length of stay.

### Effects of FNI on child ANS variables

**Fig 2:**
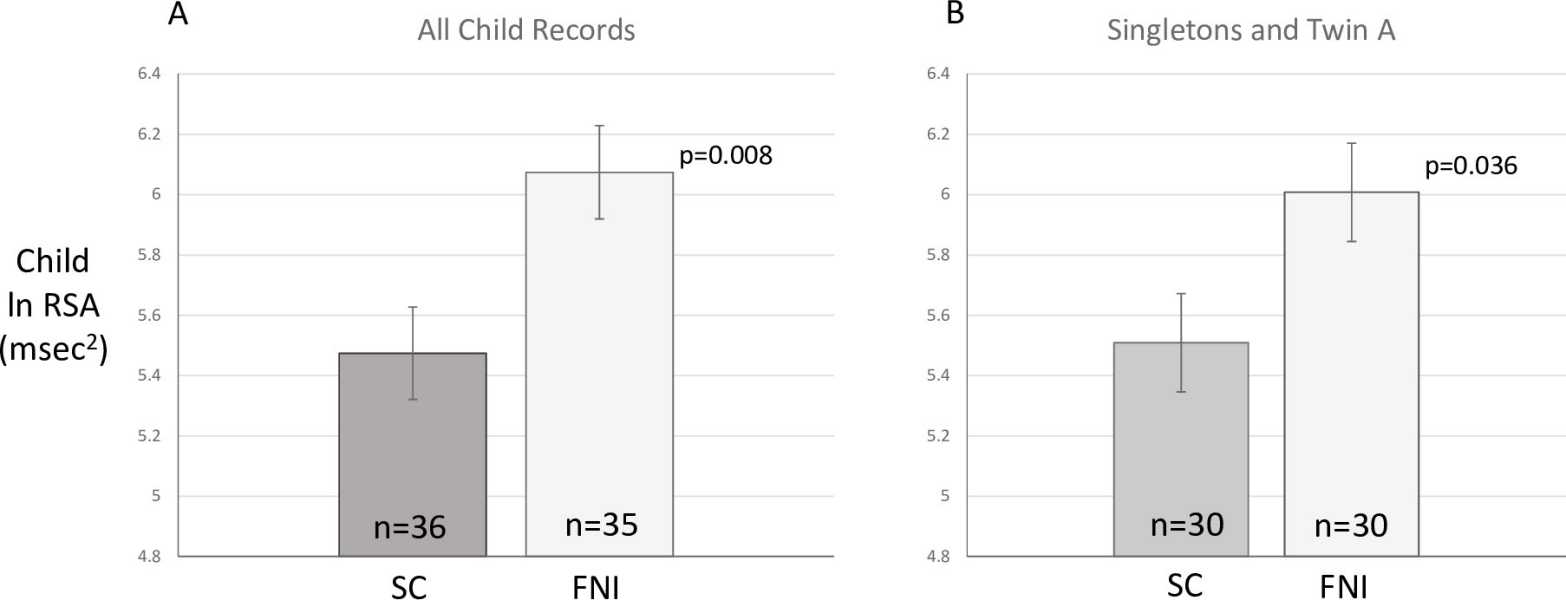
Means (±SE) of RSA for children at the 5-year follow-up during the lap-test. The set of bars on the left show results for all children and the set of bars on the right show these results when including only singletons and twin A.

Fig 2 presents the means (±SE) of RSA for children at the 5-year follow-up during the lap-test. The analyses of covariance showed that FNI infants had significantly higher RSA regardless of which strategy was used (All records: mean difference = 0.60, 95% CI 0.17 to 1.03, F(1,64) = 7.53, p = 0.008; Singleton + Twin “A”: mean difference = 0.50, 95% CI 0.04 to 0.96, F(1,53) = 4.65, p = 0.036).

Table 3 provides the means and SEs for the three other variables analyzed: HR, SD of HR, V_eff_. Note that the values for HR and SD-HR are based on averages across multiple 30 sec epochs. Values for V_eff_ are derived from 10-second epochs and reflect the slopes of the relationship between heart period (i.e., epoch mean of the R-to-R intervals) and RSA, one value for each subject. Only overall variability in HR (SD-HR) showed a significant group effect with FNI children having increased levels of SD-HR. However, when RSA is included as a covariate in the analysis of SD-HR, group differences are no longer significant (adjusted means and SEs; SC, 7.50±0.23 vs FNI, 7.69±0.23, p = 0.56). This supports the conclusion that it is the high frequency, i.e. RSA, component of total HRV that is affected by the intervention.

**Table 3:** Means (SE) for heart rate (HR, bpm), standard deviation of HR (SD-HR, bpm), and vagal efficiency (V_eff,_ bpm/in unit of RSA) for standard care (SC) and Family Nurture Intervention (FNI) children assessed at 5 years of age (60.0 months corrected age ± 6.0 months SD).

	All Records			
	SC (n = 36)	FNI (n = 35)		
**measure**	mean (SE)	mean (SE)	mean difference (95% CI)	p
**HR**	103.7 (1.4)	101.3 (1.4)	2.4 (1.55 to -6.35)	0.239
**SD-HR**	7.0 (0.3)	8.2 (0.3)	1.2 (2.05 to 0.36)	**0.018**
**V**_**eff**_	24.1 (2.0)	25.2 (2.0)	1.1 (-4.54 to 6.74)	0.716
	**Singleton + One Twin**			
	**SC (n = 30)**	**FNI (n = 30)**		
**measure**	mean (SE)	mean (SE)	mean difference (95% CI)	p
**HR**	103.1 (1.5)	102.8 (1.5)	-0.3 (-4.55 to 3.95)	0.881
**SD-HR**	7.0 (0.4)	8.1 (0.4)	1.1 (-0.03 to 2.23)	**0.038**
**V**_**eff**_	23.8 (2.2)	24.0 (2.2)	0.2 (—6.02 to 6.42)	0.944

Values for HR and SD-HR are based on averages across multiple 30 sec epochs. Values for V_eff_ are derived from 10-second epochs and reflect the slopes of the relationship between heart period (i.e., mean of the R-to-R intervals) and RSA, one value for each subject. Two sets of data are shown; the top set are values for all children, the bottom set (are values from singletons and only one twin. These means and SE are after adjustment for covariates (gestational age at birth, birth weight, age in months at the time of assessment (adjusted for weeks premature), sex, and twin status (Y/N). Abbreviations: HR, heart rate; NS, not significant; SD-HR, standard deviation of HR, V_eff_, vagal efficiency.

### Effects of FNI on maternal ANS variables

**Fig 3:**
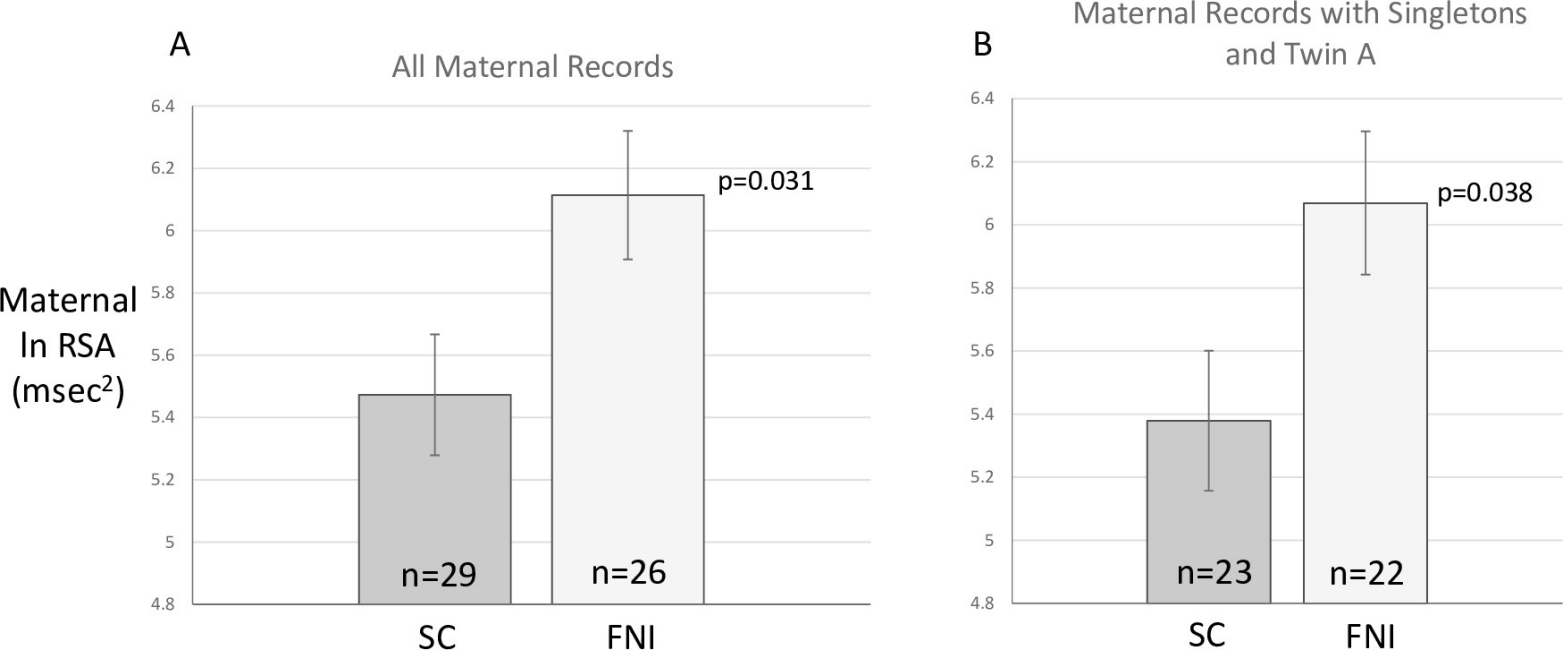
Means (±SE) of RSA for mothers at their child’s 4 to 5 year follow-up during the lap-test. The set of bars on the left show results for all children. These studies of mothers reflect multiple measures from some mothers tested with both of their twins. The set of bars on the right show results when including only singletons and twin A.

Fig 3 presents the means (SE) of RSA for mothers at their child’s 4 to 5 year follow-up visit during the lap-test. Results from the analyses of covariance showed that mothers of FNI infants had significantly higher RSA for both sets of analyses (All records: mean difference = 0.64, 95% CI 0.07 to 1.21, F(1,48 = 4.94, p = 0.031; Singleton + Twin “A”: mean difference = 0.69, 95% CI 0.05 to 1.33, F(1,38) = 4.62, p = 0.038).

Table 4 provides the means and SEs for the three other maternal variables analyzed: HR, SD of HR, V_eff_. As was the case for child variables, only the SD of HR showed a significant group difference in these three variables; FNI higher children had higher SDs than SC.

**Table 4:** Means (±SE) for heart rate (HR, bpm), standard deviation of HR (SD-HR, bpm), and vagal efficiency (V_eff,_ bpm/ log_e_ units of RSA) for mothers of standard care (SC) and Family Nurture Intervention (FNI) children assessed at 5 years of age (60.0 months corrected age ± 6.0 months SD).

	All Records				
	SC (n = 29)	FNI (n = 26)			
**measure**	mean (SE)	mean (SE)		mean difference (95% CI)	p
**HR**	73.2 (1.9)	74.8 (2.1)		1.6 (-4.06 to 7.26)	0.594
**SD-HR**	3.7 (0.3)	4.6 (0.3)		0.9 (0.05 to 1.76)	**0.035**
**V**_**eff**_	11.3 (3.1)	12.3 (3.2)		1.0 (-7.92 to 9.92)	0.818
	**Singleton + One Twin**				
	**SC (n = 23)**		**FNI (n = 22)**		
**measure**	mean (SE)		mean (SE)	mean difference (95% CI)	p
**HR**	73.0 (2.3)		76.2 (2.3)	3.2 (-3.36 to 9.76)	0.349
**SD-HR**	3.6 (0.4)		4.8 (0.4)	1.2 (0.06 to 2.34)	**0.025**
**V**_**eff**_	10.9 (3.2)		11.5 (9.3)	0.6 (-18.88 to 20.08)	0.904

Values for HR and SD-HR are based on averages across multiple 30 sec epochs. Values for V_eff_ are derived from 10-second epochs and reflect the slopes of the relationship between heart period (i.e., mean of the R-to-R intervals) and RSA, one value for each subject. Two sets of data are shown; the top set are values for all measurements made on these mothers. The lower set are values only from when mothers were tested with their singleton child or one twin. These means and SE are after adjustment for covariates (gestational age at birth, birth weight, age in months at the time of assessment (adjusted for weeks premature), sex, and twin status (Y/N)). Abbreviations: HR, heart rate; RSA, SD-HR, standard deviation of HR, V_eff_, vagal efficiency.

### Relationships between child and mother variables

In addition to analyses of mean values we also computed the relationships between child and mother variables. The partial correlations, adjusting for the same covariates as for the analyses of covariance above, are shown in Table 5.

**Table 5:** Partial correlations between child and mother lap test autonomic variables at the 4–5 year follow-up visit. Correlations were adjusted for effects of sex, gestational age, body weight, corrected age at testing, twin status on both the child and mother values.

	SC				FNI			
	N	r	p	95% CI	N	r	p	95% CI
**HR**	29	0.340	0.071	(-0.03 to 0.628)	26	0.580	**0.002**	(0.249 to 0.789)
**SD-HR**	29	0.390	**0.036**	(0.028 to 0.661)	26	0.400	**0.043**	(0.015 to 0.681)
**V**_**eff**_	29	0.020	0.918	(-0.349 to 0.383)	26	0.180	0.379	(-0.222 to 0.530)
**RSA**	29	0.100	0.606	(-.276 to 0.450)	26	0.210	0.303	(-0.193 to 0.552)

Abbreviations: HR, heart rate; RSA, magnitude of respiratory sinus arrhythmia; SD-HR, standard deviation of HR, V_eff_, vagal efficiency.

The correlation between mother and child HR was positive for both SC and FNI; although only the value for FNI reached significance (SC, r = 0.34; FNI, r = 0.58). The correlations between mother and child SD-HR were positive and significant in both groups. Neither V_eff_ nor RSA showed significant correlations between mother and child.

### Relationships between infant and child autonomic variables

In a prior publication [26] we reported on RSA and V_eff_ values obtained at ~35 weeks and at term age (~40)weeks postmenstrual age, as well for the changes in these measures between the two time-points. The results showed that early life developmental changes in RSA and V_eff_ were significantly greater for FNI infants. Table 6 summarizes correlations between these prior measures of RSA and V_eff_ and the RSA measures obtained at 4–5 years of age. These analyses showed that, across all subjects, RSA at either ~35 or ~40 weeks and in either of two sleep states (quiet sleep, QS; active sleep, AS) were significantly correlated with values obtained at 4 to 5 years of age while the children were awake and on their mother’s lap. While these correlations were positive for both SC and FNI, the correlations within groups reached significance only for FNI subjects.

**Table 6:** Correlations between RSA and vagal efficiency in infancy (~35 and ~40 weeks postmenstrual age) and RSA at 4 to 5 years of age for all subjects combined and for Standard Care (SC) and Family Nurture Intervention (FNI) subjects separately.

	All Subjects					SC				FNI			
Variable	State	N	r	p	95% CI	N	r	p	95% CI	N	r	p	95% CI
RSA_35wks_ → RSA_4-5yrs_	QS	34	**0.41**	**0.016**	(0.08 to 0.66)	19	0.28	0.25	(-0.20 to 0.65)	15	**0.65**	**0.009**	(0.20 to 0.87)
RSA_35wks_ → RSA_4-5yrs_	AS	34	**0.39**	**0.021**	(0.07 to 0.65)	19	0.29	0.23	(-0.19 to 0.66)	15	**0.60**	**0.018**	(0.13 to 0.85)
RSA_40wks_ → RSA_4-5yrs_	QS	49	**0.33**	**0.021**	(0.05 to 0.56)	27	0.27	0.182	(-0.13 to 0.59)	22	**0.45**	**0.037**	(0.03 to 0.73)
RSA_40wks_ → RSA_4-5yrs_	AS	48	**0.30**	**0.041**	(0.01 to 0.54)	27	0.21	0.306	(-0.19 to 0.54)	21	**0.50**	**0.021**	(0.09 to 0.77)
V_eff 35wks_ → RSA_4-5yrs_	QS	33	0.23	0.191	(-0.12 to 0.53)	18	0.17	0.507	(-0.33 to 0.59)	15	0.37	0.176	(-0.18 to 0.74)
V_eff 35wks_ → RSA_4-5yrs_	AS	33	0.23	0.208	(-0.13 to 0.53)	18	0.18	0.47	(-0.31 to 0.60)	15	0.43	0.111	(-0.11 to 0.77)
V_eff 40wks_ → RSA_4-5yrs_	QS	49	0.14	0.346	(-0.15 to 0.40)	27	0.25	0.217	(-0.15 to 0.57)	22	0.00	0.989	(-0.42 to 0.42)
V_eff 40wks_ → RSA_4-5yrs_	AS	48	**0.35**	**0.014**	(0.08 to 0.58)	27	**0.45**	**0.017**	(0.09 to 0.71)	21	0.30	0.188	(-0.15 to 0.65)

Abbreviations: AS, active sleep in infancy; N, number of subjects; p, p-value; QS, quiet sleep in infancy; r, correlation coefficient; RSA, respiratory sinus arrhythmia magnitude; V_eff_, vagal efficiency.

In another set of analyses, we determined whether the change in RSA from ~40 weeks to 4 and 5 years was significantly different for SC and FNI subjects (Table 6). There were 27 SC and 21 FNI subjects that had RSA at ~40 weeks and again at 4–5 years. Repeated measures ANOVAs for RSA from the infant to child time period showed significant age x group interactions (p<0.05) for data obtained during infancy in both quiet and AS states, after adjusting the ~40 week RSA values for effects of gestational age at birth, birth weight, sex, twin status, and the postmenstrual age at the term age measurement, and adjusting the 4–5 year RSA values for gestational age at birth, birth weight, sex, twin status, and the CA at the 4–5 year time point. The mean (±SE) increases in RSA during QS from ~40 weeks to those when awake at 4–5 years were +3.11±0.16 log_e_ msec^2^ for SC and +3.67±0.19 log_e_ msec^2^ for FNI. These results show that the rate of increase in RSA from infancy to childhood is more rapid in FNI subjects.

## Discussion

Over the past several decades many interventions have been designed to ameliorate the adverse consequences of premature birth. Some of these have focused primarily on reducing stress, trauma, and anxiety in mothers and fathers of these infants. In some cases, these interventions fall under the general category of Family Centered Care. The goals of these interventions is to help parents and fathers manage the challenges associated with taking care of these more vulnerable infants and, in turn, provide the infants a more supportive environment. Reviews of these interventions, support the value of such approaches for reducing parental distress and, in some cases infant outcomes of weight gain and length of stay, although effect sizes are generally modest (for review of these approaches see [30–33]. Other interventions are directed more toward the infants’ experiences in the NICU and regulation of the NICU environment. Included among these are NICCAP [34], sound reduction [35], massage [36], and skin-to-skin or kangaroo care [12]. As shown in the cited reviews, there is some evidence to support use of these interventions as ways to improve growth and neurodevelopmental outcomes of preterm infants, though again, results vary and with few exceptions (e.g. [16]) have not followed infants long term nor have jointly focused on infant and mother outcomes.

In this current report we provide evidence that a new NICU-based intervention, (FNI) alters long-term physiological outcomes of premature infants and their mothers. Specifically, we show that FNI in the NICU increased parasympathetic activity of both mothers and children at 4- to 5-years CA, when compared with mothers and children who received SC alone at the same time point. In other analyses, we combined current data with previous data analyses and found that the rate of change in parasympathetic regulation from ~41 weeks postmenstrual age to 4 to 5 years CA as measured by RSA was greater in FNI children [26]. Both of these findings extend our results at the earlier time point, which showed that FNI infants exhibit more rapid maturation of infant parasympathetic regulation from ~35 to ~41 weeks postmenstrual age. Together, these results indicate that infants who received FNI in the NICU have accelerated development in autonomic regulation from infancy well into early childhood.

In some respects, the finding that a few hours of facilitated mother-infant co-regulatory calming interactions in the NICU can lead to such long lasting changes in physiology may seem surprising. However, extensive animal literature describes the powerful effects of early life experiences on later life behavior and physiology [37–40]. Indeed, in studies of mother-pup rearing interactions among normal laboratory rats we found that: 1)”certain types of interactions between mothers and their pups may contribute to individual differences in cardiovascular system development”, 2) “…the cardiovascular system may be shaped by experiences of early life, which are embedded in mother/young behavioral interactions”, and 3)… “that some aspects of maternal behavior may change as a function of pup characteristics” [41, 42]. In other words, evidence has shown for some time that 5-year findings of HRV in the FNI group, which followed a relatively brief calming cycle intervention between mothers and preterm infants in the NICU, are consistent with findings by Feldman et al, who studied the effect of 14 days of SSC in the NICU on autonomic function into childhood.

In the FNI study, the intervention was delivered by Nurture Specialists who were trained to assess emotional connection between mother and infant and to facilitate a connection even before SSC was possible while the infant was still confined to the incubator. Mothers in the SC group were able to engage in nurturing activities of their choosing, which included SSC, non-SSC holding and communication. FNI mothers were asked to engage in non-SSC activities including exchange of scent cloths, comfort touch, vocal soothing, emotional exchange, and SSC when feasible. With regard to the improved scores on the Bayley assessments at 18 months of age, we found effects of FNI were not dependent on the amount of SSC [25]. Therefore, while the autonomic findings are similar in both studies, the differences in intervention protocol raise some intriguing questions about the mechanism of change.

FNI was adapted for the NICU environment from an intervention previously developed by Martha G. Welch MD for older children [43, 44] and was designed to facilitate the emotional connection between mothers and infants in the NICU [17]. The intervention is based on *calming cycle theory*, which predicts that socioemotional autonomic function can be changed over time via repeated calming sessions between mother and infant [45–47]. A central tenet of calming cycle theory is that autonomic Pavlovian co-conditioning between mother and fetus during gestation and the postnatal period promotes co-regulation of autonomic states and emotional connection, which underlies improved socioemotional function over time [17, 45]. Calming cycle theory is consistent with the physiological postulates of polyvagal theory [48, 49], which describe how the autonomic states of the mother and infant can be measured via HRV, cardiac vagal tone (i.e., RSA) and vagal efficiency (V_eff_) to determine the dyad’s relational health.

Our results show increased RSA in both mother and child during face-to-face contact, which we believe supports the idea that FNI-NICU led to a healthier parasympathetic calming cardiac reflex 4 to 5 years after the intervention. Thus, our findings are consistent with two theories: polyvagal theory, which elucidates the function of the mammalian vagal signaling system and how ANS states provide a physiological scaffold for emotion regulation. Our findings are also consistent with calming cycle theory, which describes a Pavlovian learning mechanism that can be exploited to change autonomic states and socioemotional relationship between mother and infant over time through repeated calming cycles.

In this study we observed higher RSA in both FNI infants and FNI mothers, but neither infants nor mothers in the FNI group had lower HRs than those in the SC group. One possible explanation for this might be related to the level of engagement between FNI mothers and infants. Our results were obtained during a brief 10-min face-to-face “lap test” at the beginning of a battery of assessments, when the pair was instructed to engage with one another as they normally would. Although we have not coded the behavioral interactions at this 4 to 5-year time point, behavioral analysis of mother/infant interactions at 4-months CA showed differences between FNI mothers and their infants [17]. For example, FNI mothers engaged more often in sustained positive touch, followed by brief moments of negative touch, followed again with positive touch. As well, FNI infants exhibited more sustained positive vocal affect and were more likely to transition sooner from negative to positive vocal affect. If greater engagement and reciprocal “give and take” characterize the interactions between FNI mothers and children, one could reasonably conclude that FNI dyads display greater variability in motor activity, when compared with SC dyads. The higher overall variability in HR (SD-HR across 30 sec epochs) found in the current study would support that hypothesis. In as much as greater motor activity would be expected to lead to higher HRs, this might counteract the effect of greater parasympathetic activity on lowering HR.

RSA is a well-established index of efferent parasympathetic input via vagal pathways to the heart (e.g., [50]). However, measurement of ANS function provides more than a good biomarker of early intervention. Our study of FNI-NICU shows that RSA may provide a valuable on-going measure of parent-child socioemotional health as well. Our findings also provide strong support for the hypothesis that there is abundant plasticity in early ANS development that makes it a prime candidate for tracking effectiveness of early interventions via RSA. Prior studies of RSA have shown developmental stability of this measure during early development, for example, from 9 months to 3 years of age [51].The current results extend these developmental correlations to both earlier (~35 weeks postmenstrual age) and later (4 to 5 years corrected) ages. Thus, our data shows RSA to be a predictable measure of autonomic function by demonstrating both responsiveness to early environmental influences and long term individual difference in stability.

Reciprocity in mother and child motor activity might contribute to the positive correlations between mothers and children with regard to HR and standard deviation of HR. However, the correlations between mothers and children for RSA were not significant despite the significant effects of the intervention on both mother and child. This supports the conclusion that, at the group level, FNI has significant effects on both mothers and children. However, at the individual dyad level, in some cases the magnitude of the effect of the intervention on RSA was stronger for the mother than for the child while for other cases the effects were stronger for the child than the mother thus leading to a lack of mother-child correlation. Nonetheless, both calming cycle theory and polyvagal theory predict that the multiple positive prosocial, attentional, and executive function effects reported in the FNI-NICU study should be associated with increases in cardiac vagal tone (RSA) in both mother and child [52, 53]. Indeed, enhanced vagal regulation over the life span should result in multiple psychological and physiological benefits [54]. Such physiological benefits could be quite significant for the mother as well. There is considerable literature to suggest that mothers are at increased risk for developing cardiovascular disease after delivery of a preterm infant [55, 56]. Given the potentially protective influence of cardiac vagal activity on the development of cardiovascular disease [57, 58], we hypothesize that facilitating emotional connection between mothers and preterm infants will provide a buffer to maternal risk for cardiovascular disease.

### Limitations

The current findings support the conclusion that FNI during the NICU stay has effects on cardiac vagal regulation lasting for years after discharge; yet, there are important caveats to these findings. One is the fact that the lap test physiological data obtained were from subsets of 55 of 115 (48%) mothers and 71 of 150 (47%) children originally enrolled in the RCT. The most recent follow-up visit prior to 4 years was at 18 months. Given the inter-visit gap of two and a half years, a near 50% follow-up rate in a city and country where relocation is common was not surprising. However, this does raise the issue of whether the FNI and SC mothers and children who returned for this visit represent special groups of the subjects enrolled in the RCT. It is also possible that FNI increases resilience against effects of a stressful environment and group differences would be similar in the lost to follow-up subjects. Accordingly, it is not possible to answer this question with the currently available data.

Indeed, there are many possible events and circumstances that could alter the course of RSA trajectories and, in turn, the effects of FNI observed. Another of these is the degree to which the recommendation made to FNI subjects at discharge to continue calming cycle sessions at home was adhered. It is also possible that some SC mothers and infants achieved the desired emotional connection after discharge on their own, which would introduce additional variance in our results. However, there were no home visits or home intervention aspects to this trial or documentation of childcare activities within the home. Thus, we cannot determine the extent to which these factors influenced our outcomes.

The design of the FNI-NICU RCT included preterm infants with a wide range of gestational ages. Because the study had this wide range and the associated heterogeneity in maternal, fetal and neonatal medical conditions, and morbidities we included gestational age at birth and birth weight as covariates in our analyses. Nonetheless, we cannot know whether the effects of this intervention would be equal for all preterm infants.

The FNI trial design also allowed for enrollment of mothers with twins. Although it is not always clear how independent mothers with twin “A” are from mothers with twin “B”, in general, it is usually best to not consider twins as independent subjects. There are multiple strategies for dealing with this issue including regression modelling with adjustment for clustering/nesting of children within mothers or using a mixed effects model. We opted to present the results two ways, one treating twins as independent samples and a second, more conservative approach of including data from only one twin.

Another limitation of this report not related to the findings per se is our understanding of how FNI can bring about such long-term changes in autonomic regulation. As noted above there are many NICU interventions tested and other new ones have been designed [59]. For all of these approaches we believe there is need to propose and test theories of change and mechanisms that would account for their effectiveness. While we have proposed such a theory of change for FNI, which includes experiencing mother infant physiological co-regulation during calming sessions, establishment of mother/infant emotional connection, and counter conditioning of adverse NICU experiences [60], we did not test this theory in the first RCT of FNI. Thus, explicating underlying mechanisms for early interventions, be they behavioral or physiological, remains a gap in the field of early intervention and preventative medicine approaches to improving outcomes in infants born too early. We are addressing this need in our ongoing replication trial of FNI that includes repeated acquisition of mother and infant physiology to better quantify mother/infant co-regulation and changes in physiological synchrony over time as an index of conditioning. In addition, we will assess emotional connection using the Welch Emotional Connection Screen [61, 62] to test the hypothesis that emotional connection underlies effectiveness of the intervention.

## Conclusions

This follow-up study of our previous FNI-NICU trial cohort at 4 to 5 year suggest that FNI may result in healthier autonomic regulation in both mother and children compared to SC. The results are consistent with other clinical and preclinical findings with respect to early postnatal mother/infant interactions and extend the research to longitudinal follow-up in childhood. These results are also consistent with polyvagal theory, which provides the evolutionary and ontogenetic underpinnings of mammalian vagal physiology in prosocial behavior, and calming cycle theory, which describes the mother and infant/child autonomic co-conditioning mechanism through which socioemotional autonomic regulation can be changed from maladaptive to adaptive. Findings support the conclusion that facilitating mother/infant autonomic co-regulation through repeated calming sessions in the NICU is a feasible and promising way to improve neurobehavioral outcomes in preterm infants.

## Supporting information

S1 Checklist CONSORT 2010 checklist of information to include when reporting a randomised trial*. (DOC)

S1 File (PDF)

S2 File (PDF)

## Abbreviations

ANS: autonomic nervous system
AS: active sleep
BW: body weight
CA: corrected age
FNI: Family Nurture Intervention
GA: gestational age
HR: heart rate
HRV: heart rate variability
NICU: Neonatal intensive care unit
QS: quiet sleep
RSA: respiratory sinus arrhythmia
SC: standard care
SSC: skin-to-skin care
V_eff_: vagal efficiency
