# Supplementary material for: Family nurture intervention in the NICU increases autonomic regulation in mothers and children at 4-5 years of age: Follow-up results from a randomized controlled trial
Source: PLoS One. 2020 Aug 4;15(8):e0236930. doi: 10.1371/journal.pone.0236930 (PMC7402490; doi:10.1371/journal.pone.0236930)
Supplement: S1 File — (PDF) [file pone.0236930.s002.pdf]

Columbia University Human Subjects Study Description Data Sheet

Protocol: IRB-AAAD0389(Y1M01) Modification | **Protocol Status: Approved** | **Effective Date: 09/24/2008**
Expiration Date: 07/01/2009

Originating Department: **PATHOLOGY (548)**
Submitting To: **Medical Center**
Title: **Enhancement of Co-regulation between Mother and Infant via Family Nurture Intervention (FNI) in the NICU: Short and Long Term Effects on Development**
Sponsor Protocol Version#:
Abbreviated title: **Family Nurture Intervention (FNI) in NICU**
IRB of record: **Columbia University Medical Center**
IRB number used by the IRB of record: **N/A**

Affiliated Institutions: -Standard Columbia Submission
Protocol Begin Date: 06/01/2008
Protocol End Date: 06/01/2013
Previous Protocol Number: none

Principal Investigator: Martha Welch (564)

Study Description

STUDY DESCRIPTION

1. STUDY PURPOSE AND RATIONALE

New insights from developmental neuroscience suggest that multiple biochemical and physiological systems are programmed by stimulation during early development and can promote or degrade health and well-being throughout life. Animal data suggests that nurture during early development plays an important role in modifying neurodevelopment and stress adaptation that persists into adulthood. For example, high levels of nurture provided by the rat dam during the first week of life are associated with changes in gene expression of glucocorticoid receptors which determine life-long stress responsivity. Variations in maternal care permanently alter the development and expression of behavioral responses to stress throughout life (9-12). Other early manipulations of rat pups can enhance spatial learning and memory in adulthood (13).
Among those groups of humans that appear to be most negatively affected by early experiences are those who are born prematurely. The combination of underdevelopment and unnatural postnatal experiences put low birth weight infants at risk for a host of early and midlife disorders. Early in life, the adverse developmental outcomes of preterm births can include cognitive and motor disabilities (6) and autism (14-16) as well as long-term vulnerability for hypertension, diabetes, and major depressive disorder (8).

This and other research warrants the investigation of better intervention strategies that can reduce morbidity in these infants. The purpose of this current study is to compare the efficacy of two in-hospital protocols to improve developmental outcomes in preterm births. The first protocol is the current standard of care in the NICU, which includes periodic Kangaroo Care, an intervention involving mother-infant skin-to-skin contact. The second is a Family Nurture Intervention (FNI) which adds other interventions (interactive touch with vocal soothing, sustained reciprocal olfactory exposure, and family modeling and practice in comforting) and more systematically implements Kangaroo Care. The behavioral, neurobiological and clinical insights gained from this project may eventually lead to better prevention of developmental disorders, reduced mortality/morbidity and more effective clinical intervention strategies both in the neonatal intensive care unit (NICU) and after discharge. We hypothesize that the treated babies compared to infants undergoing standard care only will show better results in the outcome measures in the short term and long term (see end of section for outcome measures).

The effects of the NICU environment on the development of the preterm infant have long been a concern. Within the context of optimal medical care in NICUs, separation between the mother and the infant is inevitable. Separation between the mother and the infant involves higher depression and anxiety levels on the part of the mother (17) and less

Columbia University IRB
Approved for use until: 07/01/2009

than optimal social competencies in later development on the part of the infant (18). One early study suggested that it was the pattern and not the level of sensory stimulation of preterm infants that was important (19), and recommendations have been made to modify noise (20) and light (21) settings of the NICU to provide an environment with appropriate and adequate stimulation for the proper development of infants. Many considerations are grounded in the idea that reducing stressful stimuli and approximating, as much as possible, the in-utero environment is key for optimal neurobehavioral development in the preterm infant (22, 23).

A necessary consequence of admission to the NICU is the separation of the infant from the mother and family. The time after birth that a preterm infant spends without the mother's input and regulation at a critical period of development presents an issue for both mother and child that must be addressed. Infants depend on environmental cues to regulate neurobiological and behavioral systems such as autonomic, visceral, motor, state, and the attention span (24-26), and especially the dyadic co-regulation between infant and mother (27) , which is activated after birth as an ecological interactional social system within the dyad (28).The interactive co-regulation between the mother and the infant shapes both maternal and the infant behavior. Intervention involving the mother and the infant within the early development in the NICU predicts better development (18). It has been shown that mothers' visiting their preterm infants in the NICU has important consequences for the development of their infants and for the mother-infant relationship, affecting length of stay and mother's view of the infant (29). Also, there is evidence that interactive maternal behaviors can influence the cognitive resilience at 8 months of preterm infants exposed to early life stress (30). One major barrier to interactive maternal behavior is the mothers' feelings. Her feelings make synchronous and contingent interaction difficult, and can reduce the positive effect a mother has on her infant. Our aim is to empower mothers to have a more proactive role in the development of their infants by restoring their view of the child, establishing a strong mother-infant bond and improving feelings of parental efficacy.

2. STUDY DESIGN AND STATISTICAL PROCEDURES.

Past studies have focused solely on the effects of interventions on mothers or on infants. This proposal simultaneously studies mothers and infants. While comprehensive, the proposed study is designed to provide the basis and preliminary results for more extensive future studies. In this study, infants will be randomly assigned to one of two groups. Both groups will receive standard NICU medical care. Standard NICU care includes periodic kangaroo care, psychosocial support and parent teaching. A weekly parent support group encourages parents to express questions and concerns. Parents are met with individually and, when necessary, are referred to appropriate services in the family's home community. Additionally, parents are taught about the NICU experience, the roles of the various NICU staff caring for their infant, and infant care and development. The two study groups will differ with regard to the degree to which mother/infant contact and nurturing is implemented and the amount of instruction they will receive about parenting.

As is part of standard care, if a mother appears to be suffering from postpartum depression, she will be referred to the unit psychologists, who will determine if further action is necessary and pursue appropriate care in their community.

The CONTROL group will receive, from the nursing staff, current standard of care with regard to instruction in basic baby care as well as encouragement to engage in mother/infant skin-to-skin contact (a modified implementation of Kangaroo Care). The INTERVENTION group will, in addition to the standard care, be guided by dedicated Nurture Specialists through an enhanced program of mother/infant interactions which include kangaroo care and are described below.

Nurture Specialists are research assistants who are, or have the training of Child Life Specialists (equivalent to a Masters Degree). The Specialist may also be a intensive care unit nurse, and they will be have been thoroughly trained by the responsible investigators in all aspects of the study protocol. The Nurture Specialists will be trained to carry out the added interventions which are not part of standard NICU care and will be monitored and supervised along the length of the study. While in the NICU, the Nurture Specialist will defer to the clinical staff regarding the medical care of each infant.

In order to estimate the number of subjects required for this study we make the assumption that the effect size of our

Columbia University IRB
Approved for
use until: 07/01/2009

multifaceted intervention, which includes enhanced guidance in and encouragement of Kangaroo Care as compared to current standard of care, will be similar in magnitude to that found for Kangaroo Care alone. Based upon a power analysis, it was determined that one hundred fifty mother-infant pairs would be sufficient to obtain 80% power for producing a significant difference in length of hospital stay. Previous studies that have shown a decreased length of stay with just kangaroo care as an intervention (31-34) have used subject populations of n= 74, 66, 30, and 28. To be conservative, we will have n=75 in each group to ensure statistical significance on a more acute population (lower average birth weight and age) and to allow for attrition.

Additional power analyses for effects on 6 month follow-up measures of maternal sensitivity and Bayley Mental Developmental Index based on studies conducted by Feldman et al. (35) confirmed that 75 per group will likely provide greater than 80% power for detecting a significant effect of our intervention. Furthermore, the literature shows that examining the effect of kangaroo care in a subject group of n=206 produced highly significant ($p<0.0001$) changes in the rate of weight gain (36). However, even numbers of n=28 group were sufficient to produce a significant ($p<0.05$) change in weight gain (34).

3. STUDY PROCEDURES

A schedule of study procedures is attached (refer to excel document entitled "Study Procedure Schedule". The schedule is based on the assumption of a minimum hospital stay of four weeks.)

We will conduct a pilot study and recruit five non-randomized patients to finalize logistical details for the study. All patients will be assigned to the intervention group to perfect scheduling and team member communication and ensure that the proposed procedures run successfully. The protocol poses minimal risk to subjects, and the chances of receiving the benefit of the intervention are increased, as no subjects will be assigned to the control group. In addition to the standard inclusion and exclusion criteria for recruitment, mothers must be English-speaking so as to minimize complications for the pilot study. Using a non-representative sample will not compromise the study because the data collected from these patients will not be included in the final statistical analysis.

Within a few days of recruiting eligible infants (born at 25-32 weeks gestational age at birth, see Subjects section) and obtaining informed consent (see Recruitment section), mothers in both groups will meet with the study coordinator for about 45 minutes. At this time, general demographic and SES data will be obtained. Then a schedule of meetings, about 45-60 minutes each and held at the infant's bedside or in one of the NICU family rooms, will be tailored to the mothers' expected days and times for visiting. These meetings will be devoted to the administration of one or more of the study questionnaires and will occur continuously throughout the study to maintain contact with the mothers in the CONTROL group.

Additional Procedures.

Mothers in the INTERVENTION group will be introduced to the Nurture Specialists. The Nurture Specialists will describe and emphasize the parents' unique role in their infant's development, the critical importance of bonding in the first days and weeks after birth and the importance of consistent parental presence and involvement with the infant during their infant's NICU stay. They will then summarize the elements of the intervention (see A-D below) and set up a tentative schedule of daily visits of at least one hour. If the mother can not come every day, the most frequent schedule possible will be determined. With approval of the medical staff, the enhanced program of interventions will commence with the Comfort-Touch with Vocal Soothing and Maternal-Infant Reciprocal Odor Exposure facets of the program as described below.

A) Initial Contact: Mother's Comfort-Touch with Vocal Soothing.

Rationale: Recently, it was found that dyadic reciprocity was predicted by the frequency of affectionate touch, defined as holding and passive touch, but not by stimulating/vestibular or instrumental/matter-of-fact touch (28).

Often preterm infants are not in stable enough condition for holding practices to be initiated. However, in most cases

Columbia University IRB
Approved for
use until: 07/01/2009

the mother can hold the hand of the infant without de-stabilizing the infant. The Mother's Comfort-Touch intervention extends that practice by teaching the mother to gently touch a hand or foot of the infant while engaging in soothing baby-talk. The neonate's preference for the maternal voice suggests that the period shortly after birth may be important for initiating infant bonding to the mother (37) and can, during this intervention, be used to positively reinforce mother's touch. The purpose of training the mother to deliver comforting touch is to positively condition the infant to the mother. This touch will help the infant distinguish the mother's voice (38) and comforting touch from the myriad necessary invasive procedures performed by staff and offer an already well-established active intervention that the mother can use to comfort the infant after discharge to the home. This intervention empowers the mothers by giving them a useful role in caring for their infant during this period of hospital intensive care when mothers report feeling helpless.

Method: Through a portal in the isolette to conserve thermal stability, the mother will hold the infant's hand in a gentle, comforting way. If a hand is not available (i.e. due to IVs or lines) the mother will hold the infant's foot. The mother will then speak to the infant in her native language with a soothing tone, explaining to the infant that she is present to comfort and sooth him/her and that she understands the infant's distress. This interaction should occur as often and as long as possible during the mother's visit, and will continue throughout the infant's hospital stay.

B) Reciprocal Odor Exposure

Rationale: Early odor-based recognition may be an important factor in the development of the infant-mother bond (31) . When infants are exposed to salient maternal odors, they rapidly become familiarized with their mother's unique olfactory signature (32). Maternal odor has also been used to attenuate crying in babies (33). Conversely, olfaction may be an especially salient modality for maternal recognition of infants. In one study, mothers were able to identify the odor of their infant's garment during tests conducted at one to two days after delivery (39).

This intervention does not require manipulation of the infant and therefore can be implemented before the infant is medically able to tolerate touch with vocal soothing. The mother's exposure to the infant's odors can continue after the NICU visit, it is not clear the extent to which maternal odor will be accessible to the infant. Nonetheless, there is another important rationale for this intervention. The request for the mother to bring a breast pad or another piece of clothing to each NICU visit may serve as an important motivation for the mother to attend the NICU more often by providing her with an activity that may be directly involved in her infant's well-being. She will understand that she is supplying a unique intervention that only she can provide.

Method: The mother will be asked to bring a breast pad or, if nursing, another piece of clothing that she has worn for several hours prior to her NICU visit. The mother will place the breast pad in the isolette close to the infant when she arrives so that it is present during the time that the mother is touching and vocally soothing her infant. The mother will be given, to take home, an article of clothing (cap or sock) that the infant has worn. She will be instructed to keep this article in close contact until her next visit. At the next visit, the mother will take another article that was worn by the infant while she was away. This odor exposure intervention will begin as soon as possible and continue throughout the infant's stay, even after other interventions have been implemented.

C) Kangaroo Care with Comfort Cycle Instruction

Kangaroo Care.

Rationale: Kangaroo Mother Care is a well-known and commonly practiced technique that involves skin-to-skin contact between a mother and infant. In its original form, this procedure was maintained throughout the day and served as an alternative to the NICU isolette for thermal stability of the infant (40). However, short and long-term beneficial effects of skin-to-skin contact have been noted with periodic skin-to-skin experiences such as 1 hour daily sessions over a 2 week period (35, 41). This intermittent form of Kangaroo care is currently encouraged by the nursing staff in the NICU at Columbia and will be practiced to greater or lesser degrees by some CONTROL mothers. One of the goals of our intervention is to implement daily or no less than 5x weekly kangaroo care for all mothers in the INTERVENTION group. The amount of kangaroo care in the control group will be logged.

Columbia University IRB
Approved for
use until: 07/01/2009

In a social aspect, Kangaroo Care has been shown to enhance parent-infant bonding and promote parent confidence, as well as improve parental perception of their infant (42). It further affects the mother by supporting breast-feeding, as well as increasing milk production and the likelihood of continued breastfeeding post-discharge (43, 44). The positive effects of Kangaroo Care extend to the infant as well. Skin-to-skin contact has been shown to stabilize the baby's temperature, heart rate, respiratory rate, and oxygen saturation (45), increase time in quiet sleep (46) conserve energy for healing and optimal growth, and result in higher mean daily weight gain and earlier discharge home (47).

Method: Kangaroo Care will start once permission is granted from the attending physician. The mother will be instructed to sit comfortably in a chair. With the help of a nurse or Nurture Specialist, the baby will be removed from the isolette and placed skin-to-skin on the mother's chest in an upright position. The infant will be covered with open flaps of the garment or with a blanket. With continued monitoring of infant (heart rate, oxygen saturation, respiratory rate) the infant will be held in that position by the mother for at least one hour. The period of holding will be advanced with ongoing experience.

Comfort-Cycle Instruction.

Rationale: Maternal support is the main predictor of optimal self regulation. Behavioral interventions done by the mother reactivates hidden regulators of the infant's physiological and behavioral systems (1, 48),

Mothers in the NICU feel high amounts of anxiety, depression, and hostility around the time of discharge (49). They struggle not to be overwhelmed by emotions as they revisit the course of their baby's hospitalization, the moments when survival was uncertain, the pressure to cooperate with healthcare staff, avoid conflicts, and make an effort to exert a level of control over their emotional responses to the negative experiences of a preterm delivery (50).

Although mothers have been looking forward to the day of discharge, the idea of becoming the primary caretaker at home without 24 hour medical expertise is daunting. They often become laden with anxiety about the future of her baby outside of the NICU. In addition, babies go home as soon as they are able to fully feed, but mothers often have not yet established a secure sense of competence in providing basic sustenance to her infant. Thus, mothers struggle with feeding their infant in the first few weeks after discharge and experience a period of transition before comfort develops (51). In our NICU, the mothers often give an S.O.S. call to the NICU staff within the first few days. These calls show the mother's lack of confidence in her parenting skills. They also reflect an actual lack of parenting practice and skill.

In-NICU care-by-parent experiences give the mother an opportunity to assume responsibility for her preterm infant's care and test the reality of caring for her child. Offered in the NICU setting, these experiences can help the mother to learn her infant's patterns of behavior, gain confidence in aspects of care giving and affirm her readiness for parenting at home (52). The Comfort-Cycle instruction is designed to help the mother further practice her independent caretaking skills; specifically, it will enable her to manage her infant's distress while in a supportive environment.

Flexing the baby, putting the hand on the back and raising the head and back, providing sucking, as well as soothing and cooing relax the baby (22, 53). Mothers tend to give up very quickly and turn then to a nurse when they cannot comfort their infant. Using terms of timing supporting self regulation, the mother will be helped to understand fuss-to-sleep cycles. If the father or grandmother is present, they too will be instructed in soothing techniques for co-regulation with both mother and infant.

Since NICU babies are at times too flaccid to manage, easily aroused, and difficult to comfort, the most important care giving activity that the mother must master is achieving mother-infant synchrony. Synchrony facilitates infant self-regulation and development by means of a co-regulatory process initiated by the mother (2-4). Because of the human infant's extreme immaturity at birth, infants depend on the care giving context and require specific environmental inputs to regulate biological and behavioral systems. The most important source of this input is the mother's body. Her proximity and interactive behavior externally regulate the organization of the infant's neurobiological, sensory, perceptual, emotional, physical, and relational systems (1). Optimal synchrony is difficult to achieve when the mother

Columbia University IRB
Approved for
use until: 07/01/2009

and infant are separated by the NICU experience. The Comfort-Cycle intervention is designed to teach the mother about her ability to regulate her infant, encourage the mother to attune herself to cues from her infant. and ensure that the mother is able to comfort her upset or flaccid baby.

Mother-infant synchrony is critical for easing the transition from hospital to home and facilitating infant health and development. Interventions that promote tactile stimulation of the infant and mother-infant contact have been shown to improve the infant's ability to self-regulate, and to moderate the effects of some risk factors. The purpose of training the mother in a Comfort-Cycle is to demonstrate to the mother that she is able to create mother-infant attunement, thereby modulating the stress response of the infant and transforming physiological dysregulation into autonomic, behavioral and emotional regulation.

Method: The mother's instruction in and initial exposure to the Comfort-Cycle will occur as part of her experience with Kangaroo Care. On some occasions of Kangaroo Care infants will remain calm throughout the session. However, at other times, the infant will initially be fussy or flaccid. As long as the infant remains medically stable (i.e. no severe desaturations or decelerations), this affords the opportunity for the mother and infant to be led through the comfort cycle.

The nurture specialist will instruct the mother to stand or sit in a chair and tenderly and securely embrace the infant face to face in the cradle hold breastfeeding pose, where the infant lays on the mother's forearm with the infant's belly touching the mother's. On occasions when the infant begins a session by being fussy, the specialist will then guide the mother through and explain the stages of the cycle. If the baby is flaccid, the mother will be instructed to flex the infant's limbs and talk or sing softly.

- Recognition of infant dysregulated condition. Mothers will be helped to recognize signs of discomfort or irritability in their infants. These signs are age-dependent and may be, but are not limited to, changes in facial expression, flexion or extension of limbs, and crying.

- Simultaneous distress of mother and infant. The mother can become distressed by her infant's discomfort or irritability. The specialist will encourage the mother to tolerate the infant's dysregulated state without becoming disorganized or aversive, and to persist in offering comforting embrace and soothing.

- Resolution of dysregulation. After a few minutes, the infant should calm down and be comforted by the mother's embrace and voice. The mother will be instructed to caress and kiss the infant, and verbally soothe the infant by cooing. She will then be asked to express her positive feelings about this interaction.

- Mother-infant Synchrony. By this stage, the mother and infant have both calmed down. The mother has successfully comforted her baby and enjoys embracing the baby. The baby will show age-appropriate signs of synchrony, which will be markedly different from the dysregulation which occurred at the onset of the cycle. The mother will be guided by the nurture specialist to a positive awareness of how she was able to eventually calm her baby. The nurture specialist and mother will review together the signs of distress and synchrony in the baby, the feelings of distress and despair that the mother may have felt and how she was able to withstand her own anxiety and soothe her infant.

This may continue for up to 30 minutes, and should not be interrupted for as long as possible.

D) Family Participation and Support Session: Role definition for Comfort-Cycle and Maternal Support (family as defined in exclusion criteria)

Rationale: As with the mother, close family members (fathers, grandparents, other family members in the mother's household) are anxious about how to care for the fragile prematurely born infant upon discharge from the hospital. By discharge, mothers in the INTERVENTION group will have received extensive training and guidance in how to care for their infant. It is important that family members witness the mother's competence in caring for the infant and learn that they too can participate in this care.

Columbia University IRB
Approved for use until: 07/01/2009

Method: Participating family members will be encouraged to learn about the Comfort-Cycle in order to support the procedure at home, both assisting the mother and (where necessary) performing it themselves in the mother's absence. During these sessions at the bedside, family members will witness the mother's behavior during the cycle and be instructed in comforting the mother as she goes through the process of engaging emotionally with the infant.

Near the time of discharge, a final family Comfort-Cycle session will be scheduled in the NICU Launchpad facility. Alternatively, the session may be held in one of the NICU family rooms. This session will involve the mother and as many family members as possible. At least 2 hours will be allotted to complete this procedure.

While the mother holds the infant and goes through the stages leading to synchrony and calmness, the mother will be instructed to review her trajectory of feelings since the birth and express to family members, to the infant, and to the staff her own feelings about becoming a mother and about her infant's distinct characteristics. She may also express her wishes to have the infant respond to her soothing. (These communications should be in the mother's native language). She will also be encouraged to express her hopes and dreams about their time at home and about the infant's future. She also will be asked to share with her family how much she struggled and how much she still needs her family's support as she brings her baby home.

E) Parenting Instructions for the Home

Rationale: Giving parenting instructions for the home addresses the need to prepare parents for the shift in care-taking responsibilities from the NICU staff to the parents at discharge. Parents have little practice taking care of their infant in the unit. The purpose of this intervention is to instruct parents, before they get home, on how to carry out everyday caretaking, address potential difficulties, and overcome them. We hope to strengthen the parents' sense of responsibility for their infant in a healthy way while reducing their over-protectiveness and worry about their infant's fragility.

Method: Before discharge, the nurture specialist will discuss with parents the activities that accompany becoming the primary caretaker of their infant. These experiences include dressing, bathing, changing, feeding, and taking the temperature of their infant. Practices and techniques will be reviewed and parents will be encouraged to perform all possible tasks as much as possible in the unit to get accustomed to handling their baby's needs. The nurture specialist will also promote each task as a positive bonding experience, and show parents the many ways they can interact and play with their infant during the caretaking experience. Furthermore, the nurture specialist will go over what their preterm infant's cues and milestones are, and how they might be different but are just as important as those of a full term infant.

The specialist will prepare parents to use the high nurture interventions they learned in the unit as a solution for non-medical problems they may encounter along the way.

A home guide to family nurture booklet will be a short, simply-written take-home manual published in English and Spanish that will reinforce the use of a daily Comfort-Cycle as a prevention or solution to infant distress and provide concrete ways for mothers and their families to calm their distressed baby. The guide will help to reassure parents that their concerns are normal and manageable. It will also remind parents that the comfort cycle will lead to a synchronous relationship that can help to calm the mother in addition to the baby. The book will be illustrated with photographs of the mother and baby, which will help personalize the manual and make it feel more relevant to each family. The nurture specialist will guide the mother and father through the booklet and discuss the contents as needed before discharge. As the book will include contact information for the study personnel, the families will gain continued access to and assistance from the nurture specialist. This will foster in the families a greater connection to the project and make them more likely to participate in follow-up.

Primary Dependent Measures

All infants: Prior to Discharge

Columbia University IRB
Approved for use until: 07/01/2009

- Severity of illness score (described in Study Questionnaires section)
- Weight gain (weekly average), breastfeeding at discharge (Y/N)
- From 60 minute sleep/physiologic studies: state distribution and organization, respiratory rate, O2 saturation, apnea, heart rate, cardiac rhythm, vagal tone, sleep wake states, EEG (power and coherence)
- Movement activity from actigraphy
- Neurobehavioral data from APIB and the Naturalistic Neurobehavioral Assessment

All infants: Follow-up at 42 weeks, and 6, 12, 18 and 24 months (corrected for prematurity)

Medical health records (re-admissions, etc.)
Growth, morphometrics (head circumference, height)
Breastfeeding (Y/N)
APIB and Naturalistic Neurobehavioral Assessment (at 42 weeks post menstrual age)
Neurologic examination (at 6, 12, 18, and 24 months corrected age)
Bayley-III (at 18 months corrected age)

All mothers: Prior to Discharge

- Maternal medical measures
- Family visiting patterns
- Medical history and general demographic data
- Assessments/Interviews/Questionnaires: Beck Depression Inventory (BDI-II), Postpartum Depression Screening Scale (PDSS), State-Trait Anxiety Inventory (STAI), Myself as Mother and My Baby Scale, Yale Inventory of Parental Thoughts and Actions (YIPTA)

All mothers: Follow-up

- Assessments/Interviews/Questionnaires: Parenting Stress Index (PSI)

Measures defined

- Sleep wake states. Actigraphy provides data on the maturation and consolidation of the sleep patterns (Ferber et al., 2002) and is a useful tool that has advantages over many methods of sleep/wake assessment in that it provides a noninvasive, continuous assessment that can be used for prolonged periods of time in a variety of situations (1). It can record infant sleep and wake patterns (2). Several studies have used actigraphy to look at the sleep/wake activity of premature infants in a neonatal intensive care unit (3). An actiwatch (model AW-2, Mini Mitter Respironics, Bend, OR) will be placed on the infant's ankle for the duration of the measurement and taken off when the measurement has been completed. Our actigraphy measurement will be taken at 36 weeks postmenstrual age, when the infant will have developed enough to handle the burden of a watch on the wrist or ankle. Previous studies have performed actigraphy on infants as young at 32 weeks, but testing older infants may provide more meaningful data as sleep state consolidation occurs and infants progressively adapt to a circadian activity-rest pattern that is more similar to full term infants (4). Starting at 8PM, the infant will have 24 hours of activity data recorded. The actiwatch should not interfere with the equipment surrounding the isolette. Data collected from the watch will be given a coded identifier to protect the patient's privacy. Data will be transferred from the Actiwatch to a computer using an ActiReader and analyzed using Actiware® software (Mini Mitter Respironics, Bend, OR).

- Vagal tone (High Frequency Heart Period Variability). The 60 minute sleep state physiologic recording sessions will be performed by the co-investigators at 36 weeks postmenstrual age or the week of discharge, whichever occurs first. Using data obtained during the session, we will characterize various parameters of cardiac function and regulation including assessments of parasympathetic modulation of heart rate (vagal tone). Individual differences in attention and emotional regulation are correlated with these parameters. Measures of heart rate variability and heart rate movement coupling have emerged as the primary candidates for assessment of the maturation of autonomic control during the

Columbia University IRB
Approved for
use until: 07/01/2009

perinatal period. Our working hypothesis is that these differences emerge during fetal development often as a result of in utero exposures and genotypic interactions. NICU exposures occurring at the same post conceptional ages in the premature infant may also alter the development of autonomic control. We will use data acquisition and analyses techniques that have been employed for many years in both non-human primate fetal research as well as studies of prematurely born infants (49-54).

Heart rate and respiration (either from analogue outputs from standard clinical monitors or specially designed hardware utilized routinely in our studies of human infants: DATAQ) will be digitized at 500 samples/sec and 20 samples/sec respectively. The digitized recordings of ECG and respiration will be processed using software designed specifically for displaying, marking, and analyzing data from these records. A research assistant, blind to group assignment, will view these records and, using an automated peak/trough marking routine embedded in the display program, will then mark each R-wave and the peak of inspiratory activity for each breath for the entire record. Each mark will be checked visually for accuracy and corrections necessary in the placement of these marks will be made manually.

After marking R-waves and breaths, the program computes the following parameters: mean breath to breath interval, and from that, mean breathing rate (in breaths per minute); mean R-to-R interval, and from that, mean HR (in beats per minute); standard deviation of the R-to-R intervals (SD-RRI, in msec); the square root of the mean of the squared differences in successive R-to-R intervals (rMSSD, in msec); and high frequency HPV (HF-HPV, in msec2) derived from spectral analyses. HF-HPV and rMSSD are highly inter-correlated measures of high frequency heart rate variability in the frequency and time-domain respectively and are taken as indirect indices of parasympathetic modulation of heart rate (57). For HF-HPV, spectra are calculated on each 60 second epoch using an interval method for computing Fourier transforms similar to that described by DeBoer (58). Prior to computing Fourier transforms, the mean of the RR interval series are subtracted from each value in the series and the residual series then are filtered using a Hanning window and the power, i.e., variance (in mm Hg2), within the high frequency band (0.5 2.0 Hz) is summed. Estimates of spectral power were adjusted to account for attenuation produced by this filter.

- EEG. A recent study on extremely low birth weight (ELBW) infants showed that these infants, measured near-term according to post menstrual age, manifest regional differences in EEG functional connectivity as compared to term infants (Grieve et.al., unpublished data). We will test the hypothesis that electrocortical functional connectivity (quantified by coherence) of low birth weight infants (born at 25-32 weeks gestational age), who are treated with high nurture intervention, will be more similar to that of term infants than coherence measured in a matched group of infants receiving standard care. The measure will be carried out by the co-investigators on the week before or week of discharge. Infants will be tested in their cribs with EEG electrodes placed per manufacturers' guidelines using the wet sponge electrodes of the high density EEG sensor net (Electrical Geodesics Inc; Eugene, OR). Infants will be allowed to sleep, and their sleep state will be noted every minute using standard behavioral criteria. We require 60 minutes of data.

EEG Data Archiving and Processing. Infant EEG data will be given a coded identifier to protect the patient's privacy. The EEG data will be screened visually for movement, eye blink, and EMG artifacts. Custom software will identify electrode bridging, if any, and notch filter EEG at the power line frequency and its harmonics. Epochs with any remaining artifact will be removed from further analysis. These data will be analyzed using existing custom software written by our group in the MATLAB language.

Data Analysis

Upon completion of data collection for 40 infants. the data will be analyzed to assess our status, perform more accurate power analysis, and confirm the feasibility of the investigation. The data will also be analyzed at the end of the study. These specific hypotheses will be tested:
Specific Aim 1. Mean measures of infant illness score, days on ventilation, days on CPAP, length of hospital stay will be compared between standard care and intervention groups.
Specific Aim 2. Mean measures of infant growth, weight, feeding, and morphometrics will be compared between standard care and intervention groups.

Columbia University IRB
Approved for
use until: 07/01/2009

Specific Aim 3. Mean measures of infant state distribution and organization, respiratory rate, O2 saturation, heart rate, cardiac rhythm, vagal tone, and sleep wake states will be compared between standard care and intervention groups.
Specific Aim 4. Mean measures of infant EEG power and coherence as well as neurologic development will be compared between standard care and intervention groups.
Specific Aim 5. Mean measures of maternal questionnaires on depression, perception of parenting, and perception of infant will be compared between standard care and intervention groups. The measures will also be compared within groups, comparing data collected close to the beginning and end of the infant's NICU stay.
Specific Aim 6. Mean measures of parental visiting hours and occurrence of kangaroo care will be compared within groups.

4. STUDY DRUGS OR DEVICES

N/A

5. STUDY QUESTIONNAIRES
Infant Medical Neurobehavioral Assessments:
Assessment of Preterm Infants' Behavior (APIB). The APIB (24, 25) is a comprehensive newborn behavioral assessment with demonstrated sensitivity to differentiate among subgroups of infants of varying gestational ages and degrees of risk status assessed at 2 weeks corrected age (59-62). Furthermore, the APIB appears to be sensitive to the increase in cortical gray and white matter as well as onset of myelination in the last trimester (63). APIB and Prechtl (64) data on an independent sample of 312 preterm and fullterm newborns studied at 2 weeks corrected age yielded 8 independent factors, accounting for 68% of the variance (65). The factors described the infants' behavior along the dimensions of self regulation and attention competence; motor system organization; hypersensitivity and intensity of response; irritability and arousal; asymmetry of performance; head control; attention expressivity; and motor system self regulatory efforts. These dimensions meaningfully map the theoretical constructs of interest in the current application in terms of melatonin rhythm development and lighting intervention. The examination will be performed by the study team in the NICU at 36 weeks corrected age and again during a scheduled follow up visit at 42 weeks postmenstrual age. Testers will be blinded to the group status of the infant. The examination is performed at approximately one hour before the infant's next expected feeding, typically with the infant in sleep state. In the course of the 45 minute examination the examiner elicits various behavior responses from the infant by use of the Brazelton Scale (66) maneuvers, which are graded in increasing sensory inputs from distal stimuli in sleep to proximal stimuli with inclusion of tactile and kinesthetic components (e.g. The Moro response). Alerting and attention is assessed at a time in the course of the examination considered optimal to elicit best performance. The six 'packages' of maneuvers are scored in terms of the infant's response patterns in terms of autonomic stability, motor system modulation, state organization, attention regulation, self regulation and the degree of facilitation required by the examiner in order to assist the infant in maintaining and/or returning to balanced subsystem integration. The systems are each scored on 9-point behaviorally defined rating scales. The person performing the assessment will be trained by experienced personnel. Inter-rater reliability will be established before commencing with this assessment, and will be maintained with rechecks every 6 9 months.

The Naturalistic Neurobehavioral Assessment. Detailed information about this assessment can be found in studies performed by Ferber and Makhoul (25, 67). All observations will be carried out by two trained researchers with an established high interrater reliability. The methodology employed was derived from the naturalistic behavioral observation of the newborn infant, which is a component of the NIDCAP (53, 68, 69). The method provides a comprehensive list of 74 items of specific behaviors, spanning all neurobehavioral subsystems of autonomic, motor, state and attention regulation in their simultaneity, as exhibited by the infant at the time of observation. This examination takes about 1 hour to complete and will be performed when the infant is at 36 weeks postmenstrual age.

Behavioral items will be first combined into 'clusters' according to their appertaining to one of the five neurobehavioral subsystems. The nine 'clusters' were labeled as follows: (1) optimal respiration measure; (2) irregular respiration measure; (3) motor disorganization (MD) score; (4) visceral stress response; (5) optimal flexed movements score; (6) extension movement (EM) score; (7) facial movement score; (8) positive attention signs (PAS); and (9) negative attention signs (NAS). Within subsystem they will be further sub-grouped into regulation and disorganization

Columbia University IRB
Approved for
use until: 07/01/2009

behaviors. Each behavior's frequency within a 'cluster' will be prorated to the length of observation. It follows that each cluster included 11 and 10 observation instances, respectively, multiplied by the number of behaviors.

Bayley Scales of Infant Development (BSIDIII or BayleyIII). The BayleyIII offers a standardized assessment of cognitive and motor development for children ages 1 month through 42 months. It has a core battery of five scales three scales administered with child interaction (cognitive, motor, language) and two scales conducted with parent questionnaires (social-emotional, adaptive behavior). It has been used for clinical studies including those studying premature and small for gestational age babies. The BSIDIII has been validated against numerous assessments including the BSIDII, the Preschool Language Scale Fourth Edition, the Adaptive Behavior Assessment System Second Edition, the Wechsler Preschool and Primary Scale of Intelligence Third Edition, the Infant-Toddler Social Emotional Assessment, and the Peabody Developmental Motor Scales Second Edition. Compared to the BSIDII, the BSIDIII is easier to administer and contains child-appealing manipulatives and play-based items to facilitate assessment. Additionally, extended floor and ceiling makes it easier for clinicians to identify lower and higher functioning infants and toddlers.

Severity of Illness Score (SOI). The Severity of Illness score was adapted (70) from the Manual for Postnatal Complications Scale (71). Total days of hospitalization, dependence on medical equipment upon discharge, and some of the more severe diagnoses were included. The SOI is the sum of points for hospital days and days on oxygen (1 day equals one point). Because the infants were hospitalized at least 3 weeks, indicating that they were potentially at risk for increased interactional difficulties as hospitalization lengthened, one point per day was included in the severity of illness scoring method. An additional point was assigned for each of the following diagnoses: intracranial hemorrhage; retinopathy of prematurity; neurological abnormalities; anemia; apnea; bronchopulmonary dysplasia; hyaline membrane disease; hyperbilirubinemia; hypocalcemia; infant of diabetic mother; meconium aspiration; patent ductus arteriosus; perinatal asphyxia; persistent pulmonary hypertention of the newborn (PPHN); pneumothorax; polycythemia; respiratory distress syndrome; seizures; sepsis; tachypnea; and other diagnoses. An additional point was given for being sent home on either apnea monitors or oxygen support.

Maternal Measures:
Beck Depression Inventory (BDI-II). The BDI is a 21 item self-report rating inventory measuring characteristic attitudes and symptoms of depression in adults and adolescents aged 13 years or older (72). It is one of the most widely accepted instruments for assessing severity of depression in diagnosed patients and for detecting possible depression in normal populations. The recent BDI-II (73) was developed for the assessment of symptoms corresponding to criteria for diagnosing depressive disorders listed in the American Psychiatric Association's Diagnostic and Statistical Manual of Mental Disorders- Fourth Edition. The BDI-II generally takes about 5-10 minutes to complete and can be self-administered or be administered orally. A Spanish version is also available.
Postpartum Depression Screening Scale (PDSS). The Postpartum Depression Screening Scale (74) measures maternal/paternal depression and anxiety in a 35-item Likert-type self-report instrument with 7 dimensions: (1) sleeping/eating disturbances, (2) anxiety/insecurity, (3) emotional liability, (4) cognitive impairment, (5) loss of self, (6) guilt/shame, (7) contemplating harming oneself. This instrument takes 5-10 minutes to complete. Its psychometric properties have been shown to be reliable (75, 76) and has been used for parents with infants in a NICU (77). Furthermore, a Spanish version exists (78).

State-Trait Anxiety Inventory (STAI). The concepts of state and trait play a role in anxiety. In general, personality states may be regarded as momentary samples of emotion in a person's life which reflects their personality (79, 80). State anxiety (S-Anxiety) is an often transitory emotional state which exists at a given moment in time and at a particular level of intensity. Trait anxiety (T-anxiety) refers to a relatively stable individual anxiety-proneness which can be used to reflect individual differences in S-anxiety, e.g. perception of a situation as stressful or dangerous, and to predict the probability of S-anxiety occurring in the future. The State-Trait Anxiety Inventory (81) comprises 2 separate self-report scales of 20 items each that measure state and trait anxiety (Spanish version also available). The S-Anxiety Scale (measuring state), has been found to be a sensitive indicator of changes in transitory anxiety experienced by patients in counseling, psychotherapy, and behavior-modification programs, and has been used to assess the level of anxiety induced by unavoidable real-life stressors. The T-Anxiety scale has proven useful for identifying persons who differ in motivation or drive level. The STAI has been used in studies examining parents of

Columbia University IRB
Approved for
use until: 07/01/2009

hospitalized children, the transition to a maternal role, perception of illness severity in infants, and maternal anxiety during pregnancy and fetal attachment.

Parenting Stress Index (PSI). The Parenting Stress Index Third Edition (82) is a 101-item questionnaire (requiring 20-30 mins) that includes a total score and Parent and Child Domains. It also includes the following 13 scales: Adaptability, Acceptability, Demandingness, Mood, Distractibility/Hyperactivity, Reinforces Parent, Depression, Attachment, Restriction of Role, Sense of Competence, Social Isolation, Relationship with Spouse, and Parent Health. We will be using the short form of 36 items, which only requires 10 mins to complete. This assessment has been used in many different kinds of studies across many populations. Relevant to our investigation, this index has been used in studies of families of low birth weight babies, parenting confidence for premature infants, mothers' and fathers' interaction with their premature infant, as well as mothers of premature infants post-discharge. Internal consistency ranged from .70 - .83 and test-retest reliability resulted in .82 and .71 reliability coefficients.

Myself as Mother and My Baby Scale. The Myself as Mother (SD-Self) measures the evaluative dimension of maternal identity and maternal role attainment using a semantic differential technique. The SD-Self consists of 11 bipolar adjective pairs embedded with a 22-item, 7-point semantic differential scale. Reliability estimates using coefficient alpha for 4 samples at both 1-3 weeks and 4-6 weeks postpartum range from .72 to .87. The SD-Self has been found to correlate with mothering behavior, rs =.2 to .46 (83) as well as negatively correlate with perceived stress (r=-.53) (84) and state and trait anxiety (r=-.51) (85). The My Baby (SD-Baby) Scale is 6-item, 7-point semantic differential scale which measures the evaluative dimension of baby's attributes embedded in a 21-item scale. Internal consistency values had a median reliability of .70 (Walker, 1982). SD-Baby correlates negatively (rs=-.49 to -.60) with a concurrent measure of perceived infant difficulties using the NPI Your Baby subscale (86). SD-Baby measured at 4-6 weeks postpartum has been found to be correlated significantly with preschool behavior problems. This scale has been used in a study of mothers of preterm infants (87).

Yale Inventory of Parental Thoughts and Actions (YIPTA). Includes a 15-20 minute interview covering 10 domains of parental thought and actions. They range from anxiety about the infant to ways of relating to and thinking about the infant. The YIPTA also includes a 10-item self-administered questionnaire.

6. STUDY SUBJECTS

Potential participants will be included upon meeting the following criteria:
- Infant is between 25 and 32 weeks gestational age upon admission
- Infant's weight is appropriate for gestational age (AGA)
- Infant is a singleton or twin (matched between groups)
- Mother has at least one family member in home (e.g. significant other, mother, father, sibling, aunt, grandmother, step-parent)

Potential participants will be excluded upon meeting the following criteria:
- Infant has major congenital anomalies
- Mother has known history of substance abuse
- Mother has known severe psychiatric illness or psychosis

7. RECRUITMENT

Potential participants will be screened and identified by the study coordinator via Eclypsis. The study coordinator will contact the attending physician and obtain permission to approach the mother about participating in the study. The first point of contact with the family will be done by the study coordinator at the discretion of the attending physician. The recruitment process will include the study coordinator describing the study and getting informed consent according to HIPAA and IRB regulations. Mothers who agree to be in the study will be randomly assigned to either the CONTROL (i.e. standard of care) or INTERVENTION group by the study coordinator. All subjects will be assigned a 3-digit coded identifier (for confidentiality purposes).

Columbia University IRB
Approved for
use until: 07/01/2009

8. CONFIDENTIALITY OF STUDY DATA
At recruitment, a subject will be given an identifying study number. All forms will be identified by this study number rather than the subject's name. Once information is transferred from Eclypsis to the study database, the program used by healthcare providers to record data, any information entering our database will also be identified with a study number rather than the subject's name. Access to the coding information and Eclypsis will be limited to the study coordinator and the person assigned to building and maintaining the study database. The information provided to the data analyst will be coded with the subject's identifying number.
9. POTENTIAL RISKS

It has been found that inadequate infection control measures by health care providers and mothers to children in kangaroo care units may potentially increase the risk of severe respiratory synctial virus (RSV) infection in a population group with compounded risk factors (88). Another study found a risk of nosocomial transmission of Mycobacterium tuberculosis within kangaroo care units, particularly in infectious tuberculosis-endemic areas (89). These studies were performed in Africa, where KMC is the only care available for the vast majority of premature infants. The lack of other medical care, as well as the compounded risks (e.g. HIV) which are not significant in our study lead us to believe that these outbreaks will not occur in our unit.
As mentioned above, we have considered the risks of performing kangaroo care with preterm infants. This issue is addressed in the current NICU kangaroo care protocol.

10. POTENTIAL BENEFITS

A recent review of kangaroo care discusses many of the behavioral, developmental, and physiological benefits of kangaroo care on the infant, as well as psychological and biological benefits on the parent (47). We anticipate that all the interventions will provide direct benefits to the infants by improving development and mother-infant bonding as well as resulting in earlier discharge and fewer complications post-discharge. Furthermore, the information from this study may also yield new information about the effects of maternal education and family support on the mother-infant relationship and the development of the preterm infant.
11. ALTERNATIVES
Infant massage is an alternative therapy that the mother can perform on the infant. The literature provides conflicting evidence about the effects of infant massage (90). Though newer evidence seems to be in support of performing infant massage, the literature on preterm infants is not substantial.
In addition, there are procedures such as control of external stimuli (vestibular, auditory, visual, tactile), clustering of nursery care activities, and positioning or swaddling of the preterm infant. The evidence supporting these practices is discussed in a review (23). Some of these practices, such as positioning and swaddling of the infant, are already part of the standard NICU care at Columbia. The benefits of other procedures, such as stimulation in one or more modalities, continue to be debated.
Another alternative is non-participation in this research.
(Literature Cited is attached as word document)

Columbia University IRB
Approved for
use until: 07/01/2009
