# Supplementary material for: Family nurture intervention in the NICU increases autonomic regulation in mothers and children at 4-5 years of age: Follow-up results from a randomized controlled trial
Source: PLoS One. 2020 Aug 4;15(8):e0236930. doi: 10.1371/journal.pone.0236930 (PMC7402490; doi:10.1371/journal.pone.0236930)
Supplement: S2 File — (PDF) [file pone.0236930.s003.pdf]

Welch MG, Hofer MA, Brunelli SA, Stark RI, Andrews HF, Austin J, Myers MM; Family Nurture Intervention (FNI) Trial Group. Family nurture intervention (FNI): methods and treatment protocol of a randomized controlled trial in the NICU. BMC Pediatr. 2012 Feb 7;12:14.

STUDY PROTOCOL Open Access

Family nurture intervention (FNI): methods and treatment protocol of a randomized controlled trial in the NICU

Martha G Welch[1,2,3*], Myron A Hofer[1], Susan A Brunelli[1], Raymond I Stark[2], Howard F Andrews[4], Judy Austin[4] and Michael M Myers[1,2,5], for the Family Nurture Intervention (FNI) Trial Group[1,2,5,6]

Abstract

Background: The stress that results from preterm birth, requisite acute care and prolonged physical separation in the Neonatal Intensive Care Unit (NICU) can have adverse physiological/psychological effects on both the infant and the mother. In particular, the experience compromises the establishment and maintenance of optimal mother-infant relationship, the subsequent development of the infant, and the mother's emotional well-being. These findings highlight the importance of investigating early interventions that are designed to overcome or reduce the effects of these environmental insults and challenges.

Methods: This study is a randomized controlled trial (RCT) with blinded assessment comparing Standard Care (SC) with a novel Family Nurture Intervention (FNI). FNI targets preterm infants born 26-34 weeks postmenstrual age (PMA) and their mothers in the NICU. The intervention incorporates elements of mother-infant interventions with known efficacy and organizes them under a new theoretical context referred to collectively as calming activities. This intervention is facilitated by specially trained Nurture Specialists in three ways: 1) In the isolette through calming interactions between mother and infant via odor exchange, firm sustained touch and vocal soothing, and eye contact; 2) Outside the isolette during holding and feeding via the *Calming Cycle*; and 3) through family sessions designed to engage help and support the mother. In concert with infant neurobehavioral and physiological assessments from birth through 24 months corrected age (CA), maternal assessments are made using standard tools including anxiety, depression, attachment, support systems, temperament as well as physiological stress parameters. Quality of mother-infant interaction is also assessed. Our projected enrolment is 260 families (130 per group).

Discussion: The FNI is designed to increase biologically important activities and behaviors that enhance maternally-mediated sensory experiences of preterm infants, as well as infant-mediated sensory experiences of the mother. Consequently, we are enlarging the testing of preterm infant neurodevelopment beyond that of previous research to include outcomes related to mother-infant interactions and mother-infant co-regulation. Our primary objective is to determine whether repeated engagement of the mother and her infant in the intervention's calming activities will improve the infant's developmental trajectory with respect to multiple outcomes. Our secondary objective is to assess the effectiveness of FNI in the physiological and psychological co-regulation of the mother and infant. We include aspects of neurodevelopment that have not been comprehensively measured in previous NICU interventions.

Trial Registration: ClinicalTrials.gov: NCT01439269

* Correspondence: mgw13@columbia.edu
[1]Department of Psychiatry, College of Physicians & Surgeons, 1051 Riverside Drive Unit 40, New York, NY 10032, USA
Full list of author information is available at the end of the article

© 2012 Welch et al; licensee BioMed Central Ltd. This is an Open Access article distributed under the terms of the Creative Commons Attribution License (http://creativecommons.org/licenses/by/2.0), which permits unrestricted use, distribution, and reproduction in any medium, provided the original work is properly cited.

Background

The overall goal of our program of research is to test the hypothesis that increasing the amount of specific types of mother-infant and family-calming interactions will improve neurodevelopmental and emotional outcomes in preterm infants, and behavioral and emotional outcomes in mothers.

The stress that results from preterm birth, and the requisite acute care and prolonged physical separation in the NICU, can have adverse physiological/psychological effects on both the infant and the mother [1-4]. In particular, the experience compromises the establishment and maintenance of an optimal mother-infant relationship [4-6]. These findings highlight the importance of investigating early interventions that are designed to overcome or reduce the effects of these environmental insults and challenges.

There have been numerous trials of interventions for mothers of preterm infants. However, several aspects make this current study different. Few studies have examined the effectiveness of biologically-relevant mother-infant calming activities in their breadth and complexity using an RCT design. With the exception perhaps of some Kangaroo Care (KC) or Kangaroo Mother Care (KMC) interventions, very few trials incorporate strategies that relate to mother-infant interactions while the infant is in the NICU, even fewer while the infant is confined to the isolette. No such trial to our knowledge has specifically identified the "Calming Cycle" (see Methods and Design below) as the central focus of the intervention, or included an extended family support component for the mother and infant. Few trials have included as extensive an array of physiological and psychological mother and infant outcome measures. Finally, few trials have followed the patients for two years. We seek to address these deficiencies by carrying out an RCT of a family-based nurture intervention (FNI) among the families of pre-term infants born 26-34 weeks PMA into the NICU, using blinded assessments of effectiveness with long-term follow-up.

By nurturing interactions and activities, we mean those mother-infant interactions inherently involved in mothering such as holding, touching and communicating. These activities engage the sensory systems of both the mother and her infant (i.e., sight, hearing, smell, touch, taste, temperature, vestibular, kinesthetic) [7,8]. An emerging body of preclinical and clinical evidence is revealing the critical importance of specific mother-infant interactions in regulating the physiology and behavior of the infant [9,10], and for shaping the development of their behavior and physiology [11-15]. Our intervention is designed to enlist these proximal mechanisms for the improvement of developmental outcome in premature infants.

Nurturing interactions are equally important for establishing and maintaining the mother's physiological and behavioral adaptations necessary for the care of her infant [16,17]. Based on the literature and on the extensive clinical experience of the first author, early introduction of repeated mother-infant sensory calming interactions is expected to alleviate maternal depression, anxiety, and guilt, as well as lessen infant aversion to contact that stems from separation/isolation and stressful medical procedures. These sensory calming interactions are expected to have physiological and behavioral effects on both infant and mother, including increasing the mother's feeling of competence. Once the physiology of mother and infant are altered, we hypothesize that these changes will be sustained over time.

We are combining elements of prior interventions with known efficacy and testing these within the theoretic context we call the mother-infant *Calming Cycle*. This cycle, which is depicted schematically in Figure 1, was described by the first author in the 1980's. It was subsequently refined and published as a therapeutic intervention suitable for mothers and children with a wide range of ages and disorders [18-21]. In FNI, the *Calming Cycle* procedure has been adapted for mothers with preterm infants. In so doing, we are enlarging the testing of preterm infant neurodevelopment beyond that of previous research to include outcomes related to mother-infant interactions and mother-infant co-regulation. Our outcome measures, especially those that measure mother-infant co-regulation, include aspects of neurodevelopment that have not been extensively used in a NICU intervention and never in the integrated way we propose (i.e., mother-infant physiological measures during the *Calming Cycle*).

Our intervention focuses on enabling mothers to engage in certain mother-infant interactions as early as possible after birth, within the constraints of the NICU environment. These interactions begin with odor-cloth exchange, firm sustained touch and vocal soothing, and eye contact while the infant is confined to the isolette; skin-to-skin contact and *Calming Cycle* activities are added to these as soon as the infant is able to be held and fed by the mother. The intervention also provides other family members with strategies to support the mother as she continues these interactions with her infant within the NICU and at home.

The intervention used in this trial is based on a rich history of ethological and experimental studies in animals and humans, as well as from the 30-year clinical experience of the first author treating developmental disorders. Our approach also draws on existing early

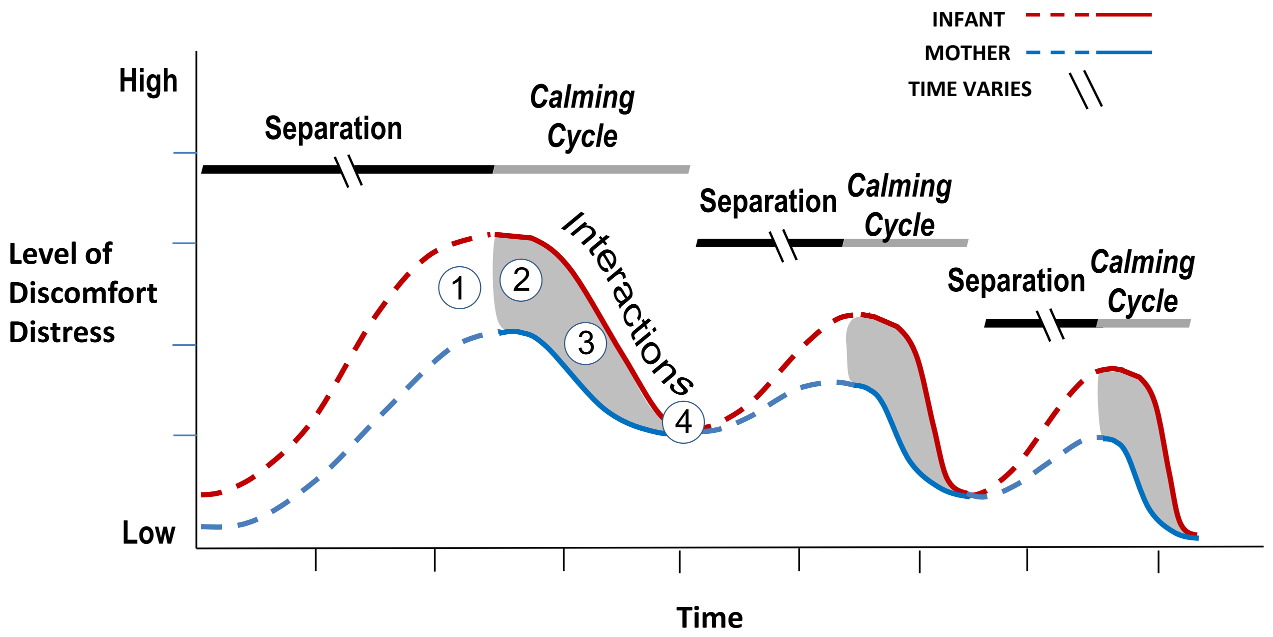

Figure 1 Conceptual representation of Calming Cycle hypothesis. During a *Calming Cycle* mothers and infants cycle through: 1) separate mother and infant discomfort/distress, 2) mutually shared distress, 3) mutual resolution of discomfort/distress, 4) mutual calm that may include periods of eye-to-eye contact and/or infant sleep. Over time *Calming Cycle* interactions will lead to more rapid reductions and lower absolute levels of discomfort and distress in both the mother and the infant. (Note: The break in the separation line indicates that in the NICU mother-infant separation times vary widely in the NICU).

intervention literature, including studies on the effectiveness of kangaroo care (KC) [22-24], Creating Opportunities for Parent Empowerment (COPE) [25,26], and Family Centered Care (FCC) [27,28].

The rationale underlying many of the features of this intervention is also supported by a recent meta-analysis of early NICU interventions involving parents [29]. The findings of this systematic review show the importance of early intervention and teaching parents skills and/or involving parents in the care of the preterm for enhancing child development. The meta-analysis focused on one primary outcome, broad neurodevelopment, which is one of the primary infant outcome measures of our trial. The prior findings support several key features of FNI: early intervention is critical, greater amounts of intervention are more effective, targeting both mother and infant is important, increasing certain types of physical contact is efficacious, and family support and involvement is of benefit.

The present report describes in detail the FNI methodology and summarizes the measures used to evaluate the efficacy, safety and practicability of this intervention. These assessments include a broad spectrum of infant physiological, infant behavioral and family health outcomes, starting from the time of enrollment in the NICU through the first two years of the infant's life. Our primary objective is to determine the effectiveness of FNI in improving the neurodevelopmental and emotional outcomes in preterm infants. Our secondary objective is to assess the ability of FNI to improve co-regulation in the mother and her preterm infant.

Methods and Design

FNI is a randomized controlled trial designed to determine the short- and long-term physiological and behavioral effects of a family nurture intervention on preterm infant development. The intervention takes place over the course of the NICU stay. FNI is designed to help the mother and her preterm infant engage in interactive biologically important activities and behaviors that enhance the maternally-mediated sensory experiences of preterm infants, as well as the infant-mediated sensory experiences of the mother. Extended family members are also engaged and encouraged to adopt both a supportive and supplementary role in the process. Our primary hypotheses are that the FNI group will have increased infant brain activity as assessed by electroencephalogram (EEG) power and better basic neurobehavioral function close to term and at 18 months CA as compared to the Standard Care (SC; described below) group. Our secondary hypotheses are that the FNI group will have decreased stress responses in mother and infant at four months CA, fewer maternal anxiety and depressive symptoms after discharge from

the NICU, as well as better maternal attachment and support systems as compared with the SC group. In addition to the aforementioned measures, we are also collecting samples of infant blood, breastmilk and stool samples from mother and infant.

Ethical considerations

Written informed consent is obtained from families prior to enrolment in the trial. This trial and all protocols within it have been approved by the Institutional Review Board of Columbia University Medical Center, where the trial is being conducted.

Participants

The study setting is a high acuity neonatal intensive care unit at Morgan Stanley Children's Hospital in New York, NY. The intervention is conducted during the infant's stay in the NICU. All follow-up assessments are conducted at adjacent facilities to the hospital at Columbia University Medical Center.

Families are eligible for recruitment if the infant is born a singleton or twin in the hospital between 26 and 34 weeks post-menstrual age (PMA), if the mother has no history of drug addiction or psychosis or other severe mental illness, if the mother understands and speaks English, and if there is at least one adult other than the mother in the home. Infants are excluded from the study if their birth weight is below the third percentile for gestational age or if there are significant congenital defects. Families are excluded if there is not at least one adult other than the mother in the home, since in such cases the critical family support component of the intervention could not be completed or evaluated (see Family Support Sessions below).

Recruitment is undertaken by a trained research assistant (RA). The RA reviews birth records daily. After obtaining permission from the attending physicians of both mother and infant, the RA invites qualifying families in the Obstetrics Ward or NICU to participate in the study. The RA describes the study procedures to the parents, including the randomization process, and obtains a written consent form from those enrolling. Mothers wishing to think about the study or talk it over with family members are encouraged to make a decision within 24 hours so that the intervention can start as early as possible.

Mothers complete initial assessments after consent and families are then assigned to the FNI intervention group or the SC group using a randomized block design. For randomization, six group assignment cards (3 FNI: 3 SC) are placed in envelopes and sealed. The envelopes are shuffled, numbered and placed in a packet prior to use. The RA withdraws an envelope from the packet to determine group assignment. Within 1 to 2 days of consenting, mothers assigned to the FNI group meet with a Nurture Specialist (NS) designated to facilitate the intervention and begin to carry this out, as described in the Intervention Methods section below. Mothers of infants assigned to the SC condition receive NICU standard care as described in the Standard Care section below. Twins are "clustered" for purposes of randomization, with both infants being assigned to the same group along with the mother.

To determine the total sample size necessary to ensure statistical power of not less than 0.8 to detect differences between group means, we considered the detectable difference for each of the primary outcome variables that would be of interest for a successful study result. Our experience and that of others suggested that effect sizes within the 'medium' range would be achievable (i.e., 0.4 to 0.6). Therefore, given the two independent samples created within the RCT design, assuming a significance level of $\alpha = .05$ for a two-tailed test, we estimate that we will have at least 80% power ($1-\beta \geq .80$) to detect a difference between groups for effect sizes and total sample sizes as described in Table 1. Experience with the pilot study suggests that we could expect attrition at a rate as high as 40%. To accommodate this, we intend to recruit a total of N = 260 participants from which a final sample size sufficient to detect all differences of interest should be achieved.

Intervention methods

Family Nurture Intervention

FNI is overseen and facilitated by specially trained Nurture Specialists (NS). Currently, these are former NICU nurses, although with proper training FNI could be facilitated by non-RNs. FNI employs three categories of calming activities that are based upon the availability of the infant for nurturing interactions with the mother and upon the availability of the family members to meet with the mother and NS. By including multiple activities, we are able to initiate at least some aspects of the calming intervention very early, when the medical status of the most vulnerable infants precludes more comprehensive approaches.

The intervention procedures in this trial are standardized and are accordingly meant to be replicable. However, the dose of the intervention varies according to the availability of the infant, the mother and the family. Mothers recruited into the study and assigned to the FNI group commit to at least four one-hour intervention sessions a week. However, FNI mothers who come in more frequently will perform these interventions more often. Research nurses facilitate at least the minimum four intervention sessions per week and keep logs of intervention activities and duration. Mothers in both

Table 1 Power analysis

Outcome Measure	PMA	Reference Population μ (σ)	Detectable Difference $\lvert\mu_{FN}-\mu_{SC}\rvert$	Effect Size d	Total N Required
			**Infant**		
**EEG** [**73**]	Term	6.044 (.434)	0.2	.46	150
**NNNS** [**88**]*	Term	4.32 (1.73)	1.0	.58	96
**HRV** [**22**]	Term	1.77 (1.10)	0.5	.45	154
**Bayley-III** [**69**]	18 months	96.4 (14)	6.0	.41	174
			**Mother**		
**Cortisol** [**89**]	4 months	Median = 2.4 (IQR: 1.6-4.1)	0.45	.40	186
**CES-D** [**53**]	4 months	10.24(9.67)[90]	5.0	.52	120
		19.8 (11.1)[91]		.45	158

A power analysis was conducted to determine the sample size needed to ensure statistical power of (1-β) = .80.

*Non-optimal reflexes. Illustrative of the 13 NNNS subscales. Citation for the reference population is the same as that for all Outcome Measures except CES-D.
Abbreviations: Bayley-III, Bayley Scales of Infant Development, III; CES-D, Center for Epidemiological Studies Depression Scale; HRV, heart rate variability; NNNS, NICU Network Neurobehavioral Scale; PMA, post-menstrual age of the infant.

groups also complete logs of weekly nurture activities. All of the nurture and intervention activities are tracked.

The first calming interactions between mother and infant take place while the infant is confined to the isolette. These activities are designed to be compatible with the limited access of the mother to the infant during the initial days in the NICU. For example, during the very first days in the NICU reciprocal odor cloth exchange may be the only way that the mother can begin to establish contact with her infant. Touching and vocal soothing are added as soon as possible. At this stage the intervention is designed to engage the sensory systems of smell, touch, hearing and sight through odor cloth exchange, firm sustained touch, vocal soothing and eye contact.

As the infant's medical status becomes stable and the infant is well enough to leave the isolette for skin-to-skin or non skin-to-skin holding and feeding, the mother is able to engage in *Calming Cycle* sessions as described below. This next stage is designed to engage the sensory systems of taste, temperature, vestibular, and kinesthetic systems in addition to the aforementioned sensory systems.

As the family members become available to meet with the NS and mother, the importance of ongoing calming activities between mother and infant as well as family support for the mother are discussed. These sessions are designed to engage the family members in reassuring and calming the mother and in providing continued support for her when the infant goes home.

Calming Interactions in the Isolette

Reciprocal Odor Cloth Exchange

Controlled laboratory experiments have shown that a mother's olfactory signature has unique relevance for her infant and that mothers recognize the characteristic scent of their newborn infant, even without extensive postnatal interactions [30-32]. Amniotic fluid, maternal breast and axillary odors are particularly salient to newborn infants. It has been demonstrated that infants orient to odors produced by their mothers and discriminate their mother's scent from odors of either unfamiliar non-parturient or unfamiliar breast-feeding women [33]. Furthermore, exposure to familiar maternal odors has a calming effect on newborn infants and can reduce crying and diminish distress [34-36]. Moreover, evidence from animal studies confirms the importance of odors for the development of mother and infant attachment behaviors and effective maternal care [37]. From this literature, we conclude that exchange of maternal and infant odors may serve as an important mode for reciprocal regulation and is thus a critical component of assessed nurture interactions. We hypothesize that regular odor exchange in the NICU during periods of physical separation, even during respiratory support, will reduce physiological and behavioral consequences of separation and facilitate the associative learning that underlies the regulation of the bond between mother and infant [36]. We encourage the odor cloth exchange even if the infant has nasal prongs and is on continuous positive airway pressure (CPAP). Although the infant may not experience the mother's odor while on CPAP, the mother is of course able to experience the infant's odor. This helps the parents connect emotionally with their infants. Moreover, the protocol for odor cloth exchange is initiated as early as possible to accustom the mother to the procedure and to promote continued use of the procedure for the entire stay in the NICU.

Procedures

During the first meeting between the mother and the NS, two small cotton cloths (approximately 5 by 7 inches) are given to the mother. On the initial visit the mother is instructed to wear one cloth in her bra and place the other cloth underneath the head of her infant,

whether or not the infant is on respiratory support. After the visit, the mother is shown how to exchange the cloth she was wearing with her infant's cloth. The mother is encouraged to repeat this cycle 1-2 times daily in the initial period of intensive care and at each visit for the duration of the hospitalization. These procedures take place within the NICU guidelines for infectious disease control. Once home, mothers are encouraged by the NS to continue odor exchanges once they are discharged from the unit during periods of separation. For example, the mother can sleep with an odor cloth and leave it with a caretaker when the mother is absent. If the infant needs soothing upon separation, he or she can then be swaddled with the mother's odor cloth.

Firm Sustained Touch, Vocal Soothing and Eye Contact

Exposure to the mother's voice begins *in utero*. Newborn term infants recognize their mother's voice within minutes of birth and prefer it to a non-maternal voice [38-40]. Yet, there is some evidence that preterm infants may not recognize their mother's voice while in the NICU [41]. This component of FNI is designed to encourage the mother to speak to her infant and respond to her infant's vocalizations in the mother's native language using varying intonations. In addition, the mother is asked to express her feelings and emotions as part of vocal and eye contact. Fathers and other family members are also encouraged to engage in similar contact and vocal soothing of the infant.

Loss of physical contact between the mother and infant can be the most salient nurture disruption that results from the NICU experience. Furthermore, much of the physical contact that infants do experience in the NICU is not calming, due to the nature of the medical procedures required. However, gentle therapeutic touch has been found to be safe for preterm infants, as measured by physiological stability and indices of agitation or pain [42]. A range of short-term beneficial effects, including maturation of visual function, attenuated pain responses, increases in vagal activity and gastric motility, and improvement in weight gain have been reported to result from touch interventions [43-46].

Procedures

During the initial meeting in the NICU the mother is instructed to make contact with her infant through firm and sustained touch and eye contact. The contact is done with sensitivity to the infant's response and physiological and emotional state. When the infant is distressed, except in the case of the smallest and most premature infants, the mother is taught to place the infant's arms on the chest and gently place her hand on top, while using her other hand to cup her infant's feet and lightly draw the knees toward the chest. The mother performs this firm tactile containment after the infant has undergone a behaviorally disruptive procedure such as suctioning, blood drawing, or even diaper change and temperature check. Once the infant is calm, the mother is taught to place her hand firmly on the infant's belly for extended periods of time as a way of providing calming sustained contact not otherwise possible while the infant remains in the isolette. The mother also is shown how to hold her finger on the tips of her infant's fingers, an action that often induces the infant to wrap his or her fingers around the mother's finger. This phenomenon, known in the literature as 'the grasp reflex', has been shown to have a calming effect on the infant [47]. Additionally, because this behavior is a form of 'answering' in response to her contact, we predict that the grasping reflex phenomenon will produce a positive emotional response in the mother, helping to establish eye contact and to reduce her anxiety and depression. This method of touching is in contrast to repeated light touching or stroking, which can be arousing and not calming [48].

While in physical contact with her infant, the mother is instructed to speak to her infant, to calm the infant, and to express her feelings and emotions. To do this, the mother is encouraged to use her primary language, the one she uses to express deep emotions. The use of the primary language has been observed to engage eye contact from the infant in a way that secondary language does not. This observation is supported by data suggesting that native and second languages are associated with distinct and different areas in the cerebral cortex [49]. It should be noted that mothers often do not know or recognize their emotional language until queried by the NS.

Calming interactions during Holding and Feeding

The intervention incorporates as a central feature repeated experience with a sequence of behaviors and states observed in clinical practice during mother and child active interaction sessions [20]. In this current paper we refer to this process as the *Calming Cycle*. This cycle consists of observable changes in the behaviors and states of both the infant and mother while they are in direct physical contact with one another for a prolonged period of time. Experience with this cycle is adapted to the varying constraints imposed by intensive care and becomes more comprehensive as infants progress medically and develop greater physiological stability and behavioral competence.

Holding sessions give the mother the opportunity to increase her engagement in the *Calming Cycle*. *Calming Cycle* experiences are encouraged to take place during feeding, skin-to-skin holding or non-skin-to-skin holding. The goal of repeated experience with the *Calming Cycle* is to enable the infant and mother to become mutually attuned to one another's physiological and

behavioral cues, and to bolster the mother's confidence in her ability to fully care for her preterm infant.

Procedures

Skin-to-skin holding is conducted according to Kangaroo Care (KC) protocols used in many studies and NICUs [48]. *Calming Cycle* sessions are initiated by the NS when the infant is off ventilator support and permission is granted by the attending physician for the infant to be outside the isolette. During the *Calming Cycle*, the mother is instructed to hold her infant safely and securely skin-to-skin and chest-to-chest, between her breasts and under her clothes, in an upright position while seated in a designated reclining chair. The infant's head is tilted up to ensure the airway is not constricted and a blanket is placed over the infant's back to help maintain temperature. Once initiated, the mother is encouraged to engage in the calming activity for a minimum of one hour, but much longer if possible.

As holding sessions proceed, the NS helps the mother identify four phases through which mothers and infants cycle: 1) separate mother and infant discomfort/distress; 2) mutually shared distress; 3) mutual resolution of discomfort/distress; 4) mutual calm that may include periods of eye-to-eye contact and/or infant sleep. Often the mother falls asleep, too. The NS helps the mother recognize each of these elements of the *Calming Cycle* and learn that the process is normal. Instruction in the *Calming Cycle* is designed to help the mother recognize her infant's physiological and behavioral signs of distress and give her a tool to use in calming her infant. It is our hypothesis that over time, *Calming Cycle* interactions will lead to more rapid reductions and lower absolute levels of discomfort and distress in both the mother and the infant. To test this hypothesis we record measures of physiological data during the *Calming Cycle* (heart and respiratory rate, temperature, and cortisol) and video tape data (for motor and behavioral assessment) in both the mother and her infant.

During these sessions, the NS maximizes a mother's comfort by providing a rocking chair or designated KC chair, dimming the lights, minimizing noise from the monitor, bringing the mother water, answering questions and addressing her concerns. The NS also helps reposition the infant if the infant is transitioning to breastfeeding. In addition, the NS encourages the mother to engage in vocal soothing and mimicking the infant's noises. Fathers are also encouraged to engage in holding sessions in order to gain experience with the *Calming Cycle* with their infants.

Family Support Sessions Previous studies have shown the beneficial effects of family education and involvement for infant development and healthcare [25,28,50]. As part of standard care, family education is provided to all families of children in our NICU; however, participation is optional. FNI goes beyond these support activities by integrating the extended family into the mother-infant nurturing care activities, and by facilitating and helping to structure a family support network for the mother and infant that will be effective throughout their time in the NICU and beyond.

FNI family support sessions involve all possible family members and others outside the nuclear family who may take part in the infant's care while in the NICU and later at home. At least one Family Support Sessions is scheduled during the NICU stay, and weekly sessions are held if possible. Special sessions may be scheduled in order to include all the various family members. During the sessions, the NS leads the family through various discussions, including the characteristics and value of the *Calming Cycle*, the needs of the infant and mother, and issues of maternal confidence, expectations, fears and concerns. Special emphasis is placed upon the mother's needs in the NICU and the ways in which extended family members can support her, as detailed in the Mom's Support Circle (Additional File 1). Issues surrounding infant care are also addressed, such as how to identify signs of infant distress and patterns of normal development.

The NS also discusses how to prepare for taking the infant home from the NICU. Practical planning issues such as where the infant will sleep, who will care for siblings, and whom the mother can call in case of an emergency are addressed. Difficulties associated with bringing home a prematurely born infant are previewed with the family and possible solutions are discussed, so that the family members are prepared for particular circumstances that concern them.

Components of a high-nurture home environment are discussed. Key elements include seeking help from family members and using conflict resolution skills to alleviate distress within the family. This is particularly important in cases where the parents are separated or divorced, or where interpersonal conflicts could interfere with the special needs of the infant. The importance of unified support is emphasized and resources and referral services are reviewed.

Finally, the mothers are asked to continue to engage in FNI activities with their infants for a minimum of 2 years. Specifically they are encouraged to use olfactory cloth exchanges when they are separated from their infants, to continue *Calming Cycle* holding sessions to reduce infant distress and to continue breast feeding. Mothers are given a Baby Trekker (Pettersen Infant Products, Manitoba, Canada) front carry pack to further encourage continued close contact with their infants.

Twins Twins as well as singletons are included in the study because twinning occurs commonly in the hospital where this study is being conducted, due largely to

increased use of *in vitro fertilization* [51]. Twins will be "clustered" and not separated in the randomization process, since FNI cannot be delivered to only one twin. The challenges of nurturing twins in the context of the study protocol are discussed during sessions with family members and an individualized plan is developed and agreed upon. When possible, mothers will hold both twins at once. Alternatively, the father and mother will take turns holding the twins at the same time. Two follow-up assessments will be impacted; only one twin per family (the first born) will be assessed at the 4- and 12-month follow-up visits, since it was determined that the first mother-twin interaction will compromise the novelty of the second mother-twin interaction and the time required to film two twins in one day will be prohibitive. The treatment of twins in the data analysis will be based upon recognition that within the twin pair the intervention cannot be independently applied.

Standard Care

None of the activities in FNI described above are explicitly part of the SC protocol for staff nurses in our NICU. Standard Care is provided equally to infants in the two groups and focuses on extensive training and practice in the management of premature infant care. Major elements of SC in the NICU focus on the maintenance of infant temperature in isolette and open crib; management of respiratory and oxygen support; provision of appropriate nutritional support; prevention and treatment of infection; pre and postsurgical care; and parent education including instruction in normal infant care, diaper change, bathing, temperature checking, holding, breast feeding, car seat use, and infant CPR.

For both SC and FNI infants, the staff nurse's attention is focused on performing and documenting the SC mandated procedures. Nurses often communicate to the mother that interacting with her infant is desirable, but facilitation of these activities is left to the discretion of the nurse. Instruction in KC can occur but it is not consistently recommended. A full-time feeding specialist is employed by the NICU who focuses on optimization of infant nutrition and evaluation of competence in oral feeding. Staff nurses provide instruction in breast pumping to mothers.

Family support group sessions are held weekly, but participation is optional and not consistently attended. Social workers and family life specialists are available when needs arise and for discharge planning as requested by parents. Family involvement with infant care is identified as important, but family engagement is at the nurse's discretion. Tasks such as feeding, bathing, and diaper change take place according to a specific time schedule. In summary, mother and family involvement in SC varies widely in implementation and participation.

Assessments

Throughout the course of the study, a comprehensive series of biobehavioral assessments is conducted in both the FNI and SC infants and their families (Figure 2). These include measures to ascertain compliance and conduct of the levels of the FNI. We also monitor the attendance and activities of the SC group.

The timing of assessments in the NICU was determined both by what we are trying to measure and by an attempt to minimize the patient burden. Thus, in the case of FNI group EEG, the time points were chosen based on practicability of first assessment and time elapse of the intervention. The four and twelve month assessments were set based on prior work showing that infant attachment at one year is predicted by mother-infant interactions measured at four months CA [52].

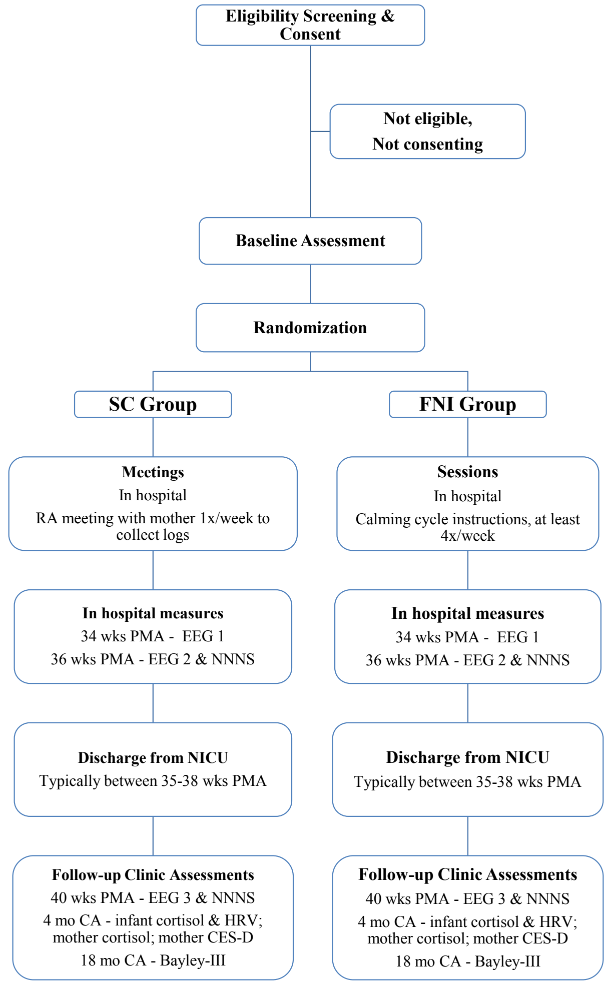

Figure 2 Study flow-chart. Abbreviations: CA, corrected age; CES-D, Center for Epidemiological Studies Depression Scale; EEG, electroencephalogram; FNI, Family Nurture Intervention; HRV, heart rate variability; NNNS, NICU Network Neurobehavioral Scale; PMA, post-menstrual age; RA, research assistant.

Other measures, such as the Speilberger State-Trait Anxiety Inventory (STAI), were scheduled to provide repeated information during the NICU stay and at follow-up time points. Many other measures were timed because of the scheduled follow-up visits. For instance, eighteen-month follow-up was chosen to coincide with the age at which the local NICU neonatologists perform Bayley III assessments. Twenty four-month follow-ups were included to capture developmental status at the conclusion of the planned study observation period.

Psychological Assessments of FNI and SC Mothers

Mothers in both groups are assessed by means of a wide-range of psychometric instruments. As summarized in Table 2 these include indices of anxiety, depression, temperament, personality, attachment, stress, and social support, that are assessed near enrolment and before group assignment and again at Term, 4, 12, 18, and 24 months CA. Prior to randomization, mothers are administered questionnaires to assess their *levels of anxiety* (Speilberger State-Trait Anxiety Inventory, STAI [53]), and *depressive symptoms* (Center for Epidemiological Studies Depression Scale, CES-D [54]). These questionnaires must be completed by the mother before her group assignment, as her responses could be affected by the assignment itself. After group assignment, the quality of the mother's attachment is assessed using the Adult Attachment Interview (AAI) [55]). The status of inhibitory vs. approach systems (Behavioral Inhibition System-Behavioral Approach System, BIS-BAS, [56]), and Mom's Support Circle [Additional File 1] are assessed after group assignment and again at Term, 4, 12, 18, and 24 months CA. Finally, questionnaires that focus on the *mother's sense of stress* (Parenting Stress Index, PSI, infant [57,58]), availability of *social support* (Norbeck Social Support Questionnaire, NSSQ [59]), efficacy as a mother (Maternal Self-Efficacy Scale, MSES [60]), and temperament (Adult Temperament Questionnaire, ATQ [61]) are administered at the 4,12, 18, and 24 month follow-up visits.

Neurobehavioral Assessments of Infants

At regular time points during the study, the neurobehavioral status of the infants is assessed using either standardized examinations conducted by research team members who are 'blind' to the infants' group assignments or through maternal questionnaires (Table 2). The first of these is the NICU Network Neurobehavioral Scale, NNNS [62], which is conducted twice, once at 36 weeks (± 1 week) PMA and again at 40 weeks (± 1 week). This assessment has several domains that provide information about the infant's neurodevelopmental status. The test measures neurological reflexes, body tone and motor maturity. It also measures the infant's orientation to and engagement with social and non-social stimuli, the lability of the infant's activity and states, and the infant's consolability. The stress/abstinence scale, originally formulated for prenatally drug-exposed infants, includes physiological, autonomic, skin, CNS, visual and gastrointestinal markers [63].

In addition to these blind assessments, mothers provide a wide range of information about their infant's development through questionnaires. At 4, 12, 18, and 24 months CA information about infant temperament is obtained using the Early Infancy Temperament Questionnaire, EITQ [64] and the Infant Behavior Questionnaire, IBQ-R [65]. Social-emotional problems of the infants are assessed by the Brief Infant-Toddler Social and Emotional Assessment, BITSEA [66] at 4, 12, 18, and 24 months CA, and at 18, 24 months CA by the Child Behavior Checklist, CBCL [67]. The mother also provides information about possible developmental delays by filling out the Ages and Stages Questionnaire, ASQ [68], at 4, 12, 18 and 24 months CA.

At 18 and 24 months CA, infants are administered the Bayley Scales of Infant and Toddler Development, Bayley III [69] consisting of four subscales: Cognitive, Language (receptive/expressive), Motor (fine/gross) and Social-Emotional. These filmed assessments, made by research staff 'blind' to infant group assignment, provide information about the neurobehavioral status of the child and whether there are significant delays in key domains of motor, language, and cognitive development.

Physiological Assessments

Physiological assessments are summarized in Table 2. The APGAR [70] score is derived after birth to give a measure of the baby's overall health. Research assistants, nurture specialists, study coordinators, and study investigators collect these measures. Blood is collected by the medical staff using the heel stick method at times determined by the clinical protocols used in our NICU. A portion of this blood is retained for use in our study. EEG is recorded using a 128-electrode net and data acquisition system (EGI, Inc, Eugene, Oregon). When possible, a one hour recording of high density EEG is obtained during sleep at two times during the NICU stay between 34 and 36 weeks PMA and after discharge at 39 to 42 weeks. ECG and respiration are also recorded during these sessions. Recordings are obtained within 30 minutes of feeding and are conducted between 11 am and 4 pm. EEG and ECG are collected at 1000 samples/sec and respiration (chest impedance belt) at 50 samples/sec. Throughout the recording sessions, RAs assign sleep state codes (quiet sleep, active sleep, indeterminate, awake, cry) once each minute, based on behavioral criteria previously shown appropriate for prematurely born infants [71]. ECG records are processed using techniques our group has developed and published previously [72,73]. These analyses provide estimates of heart rate and heart rate variability indices

Table 2 Outcome measures

Parental measures	Abbreviation		Ref	Measurement	Time(s) administered
**State-Trait Anxiety Inventory**	STAI	◆	[53]	Mother's anxiety level generally and at the current moment	E1, D, T and 4, 12, 18, 24 mo. CA
**Center for Epidemiological Studies Depression Scale**	CES-D	◆	[54]	Mother's depressive symptomology during the past week	E1, D, T and 4, 12, 18, 24 mo. CA
**Behavioral Inhibition System-Behavioral Approach System**	BIS-BAS	◆	[56]	Mother's aversive and appetitive motivation	E2, T and 4, 12, 18, 24 mo. CA
**Adult Temperament Questionnaire**	ATQ	◆	[61]	Mother's temperament	4, 12, 18, 24 mo. CA
**Parenting Stress Index (short version)**	PSI	◆	[57]	Mother's parenting stress	4, 12, 18, 24 mo. CA
**Maternal Self-Efficacy Scale**	MSES	◆	[60]	Mother's feelings of effectiveness in parenting	4, 12, 18, 24 mo. CA
**Adult Attachment Interview**	AAI	◆	[55]	Mother's perception of her intimate relationships	E2
INFANT MEASURES					
**APGAR Score**	APGAR	◆	[70]	Health of newborn children immediately after birth	1 and 5 min after birth
**NICU Network Neurobehavioral Scale**	NNNS	◆	[62]	Neurobehavioral functioning of infant	36 & 40 wks PMA
**Early Infancy Temperament Questionnaire**	EITQ	◆	[64]	Infant's temperament	4, 12, 18, 24 mo. CA
**Brief Infant-Toddler Social & Emotional Assessment**	BITSEA	◆	[66]	Screens for social-emotional problems in infant	4, 12, 18, 24 mo. CA
**Infant Behavior Questionnaire**	IBQ-R	◆	[65]	Assesses infant's temperament	4, 12, 18, 24 mo. CA
**Ages and Stages Questionnaire**	ASQ	◆	[68]	Screens for developmental delays in the infant	4, 12, 18, 24 mo. CA
**Bayley Scales of Infant Development**	BAYLEY-III	◆	[69]	Assesses infant motor, language, and cognitive development	18, 24 mo. CA
**Child Behavior Checklist**	CBCL	◆	[67]	Screens for child emotional and behavioral problems	18, 24 mo. CA
**Preschool Language Scale-5**	PLS-5	◆	[78]	Assesses toddler language development	24 mo. CA
NURTURE ASSESSMENT MEASURES					
**Mom's Support Circle (Additional File 1)**		▲		Mother's self-report of family's support system for infant care in the home.	E2, T and 4, 12, 18, 24 mo. CA
**Nurture Time Daily Log**		▲		Mother's self-report of time performing nurturing activities with the infant	Weekly in NICU
**Maternal Nurture & Family Support Meeting**		▲		Nurse's assessment of mother's nurturing ability and family support system.	1x post discharge
**Home Nurture Questionnaire**		▲		Mother's self-report of nurture interactions with her infant at home.	4 mo. CA
**Norbeck Social Support Questionnaire (revised)**	NSSQ	◆	[59]	Assesses father's social support	4, 12, 18, 24 mo. CA
MOTHER-INFANT INTERACTION MEASURES					
**Hane Behavioral Assessment Video**	HANE	◆	[76]	Coded for quality of maternal caregiving behavior and infant stress reactivity	36 wks PMA and 4 mo. CA
**Beebe Assessment Video**	BB	◆	[52]	Coded and assessed for attachment and mother-infant attunement.	4 and 12 mo. CA
PHYSIOLOGICAL MEASURES					
**Electroencephalogram**	EEG		[73]	Records brain activity of infant during an hour of sleep	34§, 36§, 40 wks PMA
**Electrocardiogram**	ECG		[92]	Records heart rhythm of infant during an hour of sleep	34§, 36§, 40 wks PMA and 4, 12, 24 mo. CA
**Respiration**			[93]	Respiratory rate of infant recorded during an hour of sleep	34§, 36§, 40 wks PMA
**Body Temperature**			[94]	Body temperature	34§, 36§, 40 wks PMA
BIOLOGICAL SAMPLES COLLECTED					
**Breastmilk**				To analyse peptides present in mother's breastmilk	3x in NICU
**Blood**				To measure cytokines present in infant's blood	Once in unit.

Table 2 Outcome measures *(Continued)*

**Cortisol**	[95]	From saliva. Assess mother and infant's stress levels	34§, 36§, 40§ wks PMA and 4, 12, 18, 24 mo. CA
**Stool**	[96]		1x/day for 3 wks, 1x/wk for 1 mo., 1x/mo. for 1 year
◆ Standardized Questionnaire		▲ Custom FNI-NICU Questionnaire	

Assessments in mothers and infants will be performed from birth through 24 months to determine the primary and secondary outcomes of this study.

§ At these approximate ages the noted physiological variables will be obtained, simultaneously, from the mother and infant during a calming cycle.

Abbreviations: CA, corrected age; D, Near Discharge; E1, Near Enrollment Before Assignment; E2, Near Enrollment After Assignment; PMA, postmenstrual age; T, At Term.

of autonomic regulation for each sleep state. EEG data are processed to obtain spatially-dependent measures of wave amplitude (power) at specific frequencies for each spatial location as well as measures of functional connectivity (coherence) between electrode locations [73].

Heart rate variability (HRV) will be assessed to provide indices of autonomic control during the three EEG assessments completed in the NICU and during the follow ups at 4, 12 and 24 months CA. HRV refers to rhythmic and predictable time-related changes in inter-beat intervals (IBIs). HRV can be seen in a continuous recording of IBIs (or heart rate) over time. It is best seen while the infant is breathing normally or breathing at a paced rate within a normal physiological range. Heart rate variability is a measure of autonomic function. Respiratory rate and variability are measured at the same times using a respiratory belt and body temperature is measured employing a temperature probe taped to the flank of the baby. These measures provide an overall indication of the infant's autonomic function.

As a component of the mother-infant interaction assessments conducted at 4 and 12 months CA (see below), salivary cortisol levels are measured at four times during each session (and additionally at 18 and 24 months CA). Previous research has shown that neuroendocrine responses measured via cortisol in pre-term infants are a robust marker of stress reactivity [74]. These measures provide physiological indices of how the infants respond to a social interaction stressor. In addition, breastmilk, saliva (mother and infant), and infant blood for peptides/cytokines/gene arrays are collected and stool is sampled to assess the gut microbiome and markers of inflammation.

Mother-Infant Interaction Assessments

Diaper Change and Feeding At 36 weeks PMA, while the infant is still in the NICU, and again at 4 months CA, videos of the mother changing the diaper and feeding the infant are obtained. These 5-10 min DVDs provide a means of assessing the qualities of the mother's interaction with her infant during a routine caregiving procedure, and are coded by research team members unfamiliar with the dyads' group assignment [9]. The mother is instructed to undress her infant, remove the diaper, wipe clean, and re-dress her infant. These familiar caregiving procedures represent a mild, ecologically valid stressor [75]. Following this diaper change, the mother is observed as she holds the infant for 15-minutes and then feeds the infant. Maternal behavior is coded during feeding and holding interactions, using a 9-point Likert scale [76] for five domains: (a) Acceptance vs. Rejection, (b) Soothing capability, (c) Consideration vs. Intrusiveness, (d) Quality of Physical Contact, (e) Quality of Vocal Contact, and (f) quality of transitions during feeding. From these data a composite score of maternal "Sensitivity" is generated.

Face-to-Face Interactions At the 4 and 12 month CA follow-up visits to the clinic, a split-screen filming session is conducted following protocols developed in the laboratory of Beebe and Jaffe [52]. These videotapes are subsequently analyzed, by team members 'blind' to the dyads' group assignments, in order to assess mother-infant face-to-face communicative competence, sensitivity of maternal caregiving and mother and infant physiological capacity to cope with a stressor. The ~60 min 4-month protocol (Figure 3) consists of the following segments: (1) obtaining mother's consent, (2) mother-infant play, (3) stranger-infant play, (4) diaper change, (5) the Still Face protocol in which mothers assume an expressionless face for two minutes [77], (6) administration of the maternal questionnaires. Cortisol is measured at 3, 12, 18 and 24 months CA (Table 2). Data from these sessions are used to determine mother and infant social competence during spontaneous face-to-face interactions and changes in interactions in response to the Still Face probe. Primary measures are: infant gaze aversion, mother-infant gaze coordination, infant distress, maternal interaction style (affectionate vs. intrusive), and responses of mothers and infants to gaze aversion.

Assessments of NICU Activities

Ascertainment of attendance and the frequency and/or time spent engaged in key aspects of the FNI are obtained from maternal self-report forms. These forms are filled out after each visit to NICU. The mother is asked to enter information about the start and stop times of each of her visits to the NICU and to estimate the amount of time or number of times she engaged in

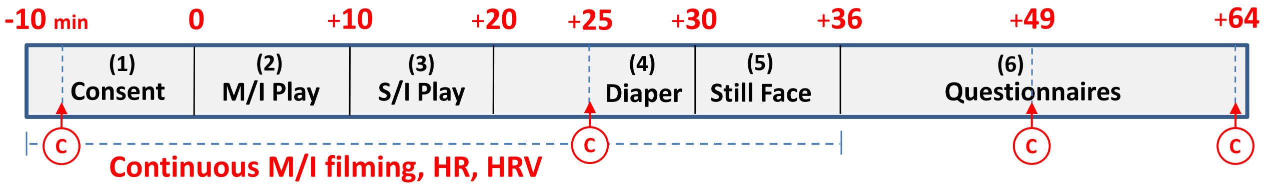

Figure 3 Four-month follow-up visit time-line. The ~60 min protocol consists of: (1) consent, (2) mother-infant play, (3) stranger-infant play (4) diaper change, (4) Still-Face paradigm, (5) parental questionnaires. Cortisol (c; "red circle") is measured at 4 times: consent, ~15 min after mother-infant play (before diaper change), 15 min after still-face (~min 49), and 30 min after still-face (~min 64). Abbreviations: c "red circle", cortisol; HR, heart rate; HRV, heart rate variability; M/I, mother-infant; S/I, stranger/infant.

various types of interactions with her infant. A similar schedule of questionnaires is given to the SC group mothers, but without questions that pertain only to the FNI group (e.g., odor cloth exchange, *Calming Cycle*). Documentation of the extent to which the SC group engages in KC, holding, and feeding will provide some information about potential cross-contamination between the groups.

Research nurses keep logs of intervention activities and their duration while facilitating the intervention with FNI mothers. Additionally, mothers in both groups complete logs of weekly nurture activities. It is possible that kangaroo care is done in the SC group and we request that the mothers log this information. It is important to note that calming touch, vocal soothing, and odor-cloth exchange are not aspects of SC in the NICU.

Primary and Secondary Outcome Measures

The primary outcome measures assess the neurodevelopment and physiological functioning of the premature infant from birth through 24 months CA as summarized in Table 2. Primary outcome measures include: infant brain activity at term, as measured by EEG; infant neurobehavioral function from response to NNNS [62], preschool language scale [78], ASQ [68] and at 18 months CA by Bayley-III Scale [69], and the stress response at 4 months CA in infants, as measured by heart rate variability (HRV) and cortisol levels.

Our secondary outcome measures assess the co-regulation of the mother and the infant. Mother's mood, temperament and attachment will be assessed using: STAI, CES-D, BIS-BAS, ATQ, PSI, MSES, and the AAI. The infant's mood and temperament will be assessed using: BITSEA, IBQ-R, EITQ and CBCL. Mother-infant interactions and attachment will be assessed through scoring video recordings. Mother-infant stress responses will be evaluated by cortisol level assessment and autonomic function recordings (HRV, respiratory rate, body temperature). A mother's nurturing ability will be assessed by self-report and NS rating of nurturing abilities and the duration of nurturing behaviors as revealed by the Nurture Time Daily Log, the Maternal Nurture & Family Support Meeting, the Home Nurture Questionnaire and the NSSQ. An additional secondary outcome is the degree to which FNI may improve family support, which is assessed with the Mom's Support Circle worksheet. We will also collect data for an additional secondary, exploratory outcome-the effect of FNI versus SC upon peptide/cytokine/gene expression in the blood and/or breastmilk and upon the gut microbiome.

Planned analyses

Descriptive statistics will be derived for all predictor and outcome variables. Family socio-demographic characteristics are not expected to differ by study group, but significance tests will be conducted to confirm the success of the randomization process. Outcomes for the two study groups including infant EEG measures, stress and development, and maternal stress and depression will be compared at each measurement interval (intake, term, 4, 12, 18, and 24 months CA) using independent t-tests for differences between means for continuous data and chi-squared tests for differences between proportions for categorical data. The Bonferroni correction will be applied to account for multiple testing. Longitudinal analyses assessing differences over time will be conducted using repeated measures ANOVA for continuous data and Kruskal-Wallace or Friedman's adaptation of this test for non-parametric data. Generalized linear models will be used to assess the variability in infant development, as measured by Bayley-III Scales at 18 months CA. This variability is explained by repeated maternal and infant psychological and neurological factors after controlling for baseline family socio-demographic characteristics.

Psychometrics

A set of well-known instruments with established validity and reliability was selected for psychometric testing in this study. For example, amongst the primary outcome variables of interest, concurrent validity with other tests of cognitive and motor skills such as the Wechsler Pre-School and Primary Scale of Intelligence (WPPSI-III) (range: r = .47, fine motor skills; r = .82 language

skills), PLS-4 (range: r = .44 motor skills, r = .69 receptive communication) and the Peabody Developmental Motor Scale (range: r = .41, expressive communication; r = .57 motor skills) have been documented, while split-half internal consistency reliability of between r = .86 (fine motor coordination) and r = .93 (language skills), and test-retest reliability ranging from r = .80 (fine motor ability) to r = .87 (language competency) has been determined for the Bayley-III Scale [69]. Discriminant validity between normal and at-risk infants [79] and significant test-retest reliability (r = .44) across three occasions [80] has been reported for the NNNS; discriminant validity (depressed vs. non-depressed) and concurrent validity (compared with clinical assessment) has been demonstrated, while internal consistency reliability has been confirmed via both Cronbach's alpha and the Spearman-Brown split-half method, with estimates ranging between 0.85 and 0.9 for the CES-D Scale [54,81]. An exception is the Mom's Support Circle (Additional File 1) measure, which was devised specifically for the NICU study and has not yet been validated (See Table 1 for power analyses).

Blinding

Data coding and analysis All data analyses are conducted on de-identified data, and assessments are made without knowledge of the mother and infant's group assignment whenever possible. Coding is performed by raters blinded to subject assignment.

Self-report assessments by mothers Mothers in the two groups read and respond to self-report questionnaires independently and receive advice and consultation that may reduce anxiety and distress. However, it is not possible to blind mothers to group assignment because mothers in the intervention group are given extensive instruction in nurture activities and are given personal attention by the NS. In the course of receiving normal NICU care and attention, mothers in the SC group may observe FNI mothers receiving supplementary attention. Moreover, the infants in the two groups may occupy nearby isolettes. Thus, SC mothers may interact with FNI mothers and observe the FNI activities.

Objective assessments Neurobehavioral assessments, including the NNNS and Bayley-III Scale, are conducted by clinical psychologists blinded to group assignment. Infant and mother physiological data (e.g., cardio-respiratory, EEG) is collected by non-blinded research assistants but coding, data reduction and preliminary data analysis is done without knowledge of group assignment. Only final group comparison analyses are, of necessity, performed with knowledge of group status.

Confidentiality of Study Data

At recruitment, a subject is given a unique study number. All forms and data are identified by this number, rather than by the subject's name. Patient data are stored in locked cabinets and access is limited to the study staff. All data collected in this study are computerized, managed and stored under the oversight of the Data Coordinating Center at Columbia University College of Physicians and Surgeons, in a physical and electronically secure relational database management system using Scientific Information Retrieval (SIR) software.

Discussion

Nurture may be viewed as the integration of multiple processes within the mother-infant dyad, from cognitive and emotional to physiological to molecular levels. The biobehavioral processes that comprise mother-infant interactions provide regulation for both members of the dyad. In very young animals and human infants, the biological systems regulated by nurture are those from which psychological/motivational and cognitive systems develop as the organism matures [82]. An emerging body of animal and clinical evidence is revealing the critical importance of specific mother-infant interactions on regulating the physiology and behavior of the infant in the short term, and for shaping the development of their adaptive behavior and physiology, extending into adulthood [82-86]. Our intervention is designed to activate these proximal mechanisms for the improvement of developmental outcome in premature infants. Changing these interactions during early development is known to have profound long-term effects on the development of the infant and subsequent maternal behavior. These changes can also have transgenerational effects [7].

The decision to include the word "nurture" in the title for the intervention in this study was made with careful consideration. Nurture has long been associated in the lay community with a wide and complex range of mother and family environmental influences that are important to infant and child development. Yet, the term nurture remains loosely defined and largely misunderstood in modern society. Perhaps because of this, nurture as the subject for serious study has come to be viewed by a large segment of the scientific community with skepticism or dismissal. Increased demand for evidence-based practice and for simple quantifiable interventions has resulted in a reductionist approach to treatments, and studies testing the effects of interventions have become simpler as data has increased in quantity and has become more complex. As a consequence, few studies have viewed the nurture phenomenon in its breadth and complexity using a multi-disciplinary approach. The purpose of this trial is to address these shortcomings of NICU intervention studies. That is, we are attempting to study the complex multi-variable nurture phenomenon by keeping focused on the biology of development and by approaching

nurture through its individual measurable components. In doing so, we hope that this study will lead to a more specific and comprehensive understanding of the word nurture in the public realm and a new appreciation of the concept in the scientific community.

For premature infants in isolettes in the NICU, we are trying to alleviate the multifaceted consequences of nurture deprivation revealed by studies of mother-infant separation in animal models [83-86]. Accordingly, we are enhancing the infant's exposure to the fundamental components of nurture, including maternal odor, touch and voice, and the warmth and tactile experiences of feeding and skin-to-skin contact. Mothers of NICU babies also experience the effects of separation; thus, our intervention is also designed to increase maternal exposure to her infant through the *Calming Cycle* interactions, with the goal of promoting biological and behavioral effects that are contingent upon normal co-regulatory interactions.

Based on the literature and on the extensive clinical experience of the first author, early introduction of repeated mother-infant sensory interactions is expected to alleviate maternal depression, anxiety, and guilt as well as lessen infant aversion to contact that stems from both separation/isolation and the many stressful medical procedures performed as life-saving interventions. The *Calming Cycle* is expected to have global and specific effects on both mother and infant. We hypothesize that repeated engagement of the mother and her infant in the *Calming Cycle* will facilitate the effectiveness of co-regulation (as measured by specific neurobehavioral, physiological and emotional indices) and have immediate and long-term beneficial effects for both. Further, we propose that the *Calming Cycle* provides a basis for helping mothers learn and integrate into their behavioral repertoire a means to initiate interactions with their infants and to respond in ways that maximize states of calm and minimize states of apparent discomfort.

Potential limitations

There are several limitations to this study. Comparing outcomes in two treatment groups with different patterns of care taking place on the same intensive care unit creates the potential for interactions between participants that can lead to unintended alterations in the treatment actually delivered and in the health of the participants, which, in turn, can affect the outcome measures themselves. For example, whereas the medical and nursing staff is explicitly blind to group assignment, they too may become aware of group status by witnessing NS activities in the FNI group. However, it should be noted that patients in both study groups are intermixed in the intensive care unit among a far greater number of non-study infants. The study NICU census averages 70 infants, with 10 being the maximum number of study infants at any one time, so that the probability of such interactions is low. However, in order to assess the possibility or extent of unintended alterations in treatment, we have included questionnaires for nursing staff and mothers as well as quantitative measures of the specific calming interactions actually taking place between the mothers and infants in both groups.

Another limitation is variation in infant health. Extensive data on infant health is recorded on the unit and differences between groups in rates of infection, medical procedures necessary, and oxygen requirement may influence the infant's availability for intervention or the outcome measures, independent of the intervention. It also should be noted that the mother's availability for spending time in the NICU is limited by other obligations, such as work or other children at home. This factor, however, should not differ significantly between groups. In summary, the NICU is such a busy place that we believe interactions between mothers in the two groups regarding the maternal care differences should be minimal.

To reduce the bias potentially associated with a randomized controlled trial of this size in a modern NICU, the following criteria developed by the Cochrane Collaboration for evaluation of intervention studies [87] will be adopted: (1) the sequence generation of allocating subjects will be random; (2) group designation will be concealed until after consent is obtained and the first set of questionnaires is administered; (3) the blinding of participants, research and clinical staff, and outcome assessments will be achieved to the extent possible; 4) outcome data will be complete; (5) outcome reporting will not be selective.

Conclusions

This FNI study will provide neurodevelopmental evidence about the importance of early and extensive mother-infant interactions, within the constraints of the NICU. This study will also test the practicability of the Family Nurture Intervention as a means to improve outcomes in prematurely born infants and their families. We are hopeful that the evidence-based methodologies employed in this study will also lead to an improved level of personalized care in the NICU, enhanced family involvement, and a more constructive relationship between the hospital and the community.

Additional material

Additional file 1: Mom's Support Circle.

List of abbreviations

AAI: Adult Attachment Interview; ATQ: Adult Temperament Questionnaire; BIS-BAS: Behavioral Inhibition System-Behavioral Approach System; CA: Corrected Age; CES-D: Center for Epidemiological Studies Depression Scale; COPE: Creating Opportunities for Parent Empowerment; FCC: Family Centered Care; FNI: Family Nurture Intervention; KC: Kangaroo Care; MSES: Maternal Self-Efficacy Scale; NICU: Neonatal Intensive Care Unit; NIDCAP: Newborn Individualized Developmental Care Assessment of Preterm infants; NS: Nurture Specialist; NSSQ: Norbeck Social Support Questionnaire; PMA: Postmenstrual Age; PSI: Parental Stress Index; RA: Research Assistant; SC, Standard Care.

Acknowledgements

Supported in part by Columbia University's CTSA grant No. UL1 RR024156 from NCRR/NIH and the Einhorn Family Charitable Trust, which had no influence upon the study design, data collection, analysis and interpretation of data, nor participated in the writing of the manuscript or submission/ publication process. We would also like to thank Rob Northrup, Erin Guerriero, Molly Arnn, Talya Lieberman and Evaline Tso for their assistance with the study and Sara Glickstein and Sue Ann Power for their invaluable help with editing the manuscript.

The FNI Trial Group includes: Ladan Afifi, David A. Bateman, Beatrice S. Beebe, Kathryn E. Carnazza, Christine Y. Chang, Patricia A. Farrell, Ewelina S. Fiedor, William P. Fifer, Phillip Grieve, Amie A. Hane, Joseph Jaffe, Jeffrey G. Johnson, Qais Karim, Shanna Kofman, Yasmine A. Koukaz, John M. Lorenz, Robert J. Ludwig, Mary T. McKiernan, David Merle, Richard S. Polin and Rakesh Sahni.

Author details

[1]Department of Psychiatry, College of Physicians & Surgeons, 1051 Riverside Drive Unit 40, New York, NY 10032, USA. [2]Department of Pediatrics, College of Physicians & Surgeons, 630 West 168th Street, New York, NY, 10032, USA. [3]Department of Pathology & Cell Biology, College of Physicians & Surgeons, 630 West 168th Street, New York, NY, 10032, USA. [4]Mailman School of Public Health, Columbia University, 722 West 168th Street, New York, NY, 10032, USA. [5]Department of Psychiatry, New York State Psychiatric Institute, 1051 Riverside Drive, New York, NY, 10032, USA. [6]Department of Psychology, Bronfman Science Center, Williams College, 18 Hoxsey Street, Williamstown, MA, 01267, USA.

Authors' contributions

All authors read and approved the final manuscript.

MGW and MMM conceived of the study, share in the responsibility for the conduct of trial and participated in the preparation of all drafts of the manuscript.

MGW designed the interventions and trains the personnel who facilitate the interventions.

MMM advised on methodology and data acquisition and participated in the preparation of all drafts of the manuscript.

MAH advised on the trial design and methodology and participated in the preparation of all drafts of the manuscript.

SAB advised on assessment measures and conducts assessments.

RIS advised on the methodology, acts as liaison with medical staff and participated in the preparation of the manuscript.

HA advised on data management and participated in the preparation of the manuscript.

JA designed and performed statistical and power analyses and participated in the preparation of the manuscript.

The FNI Trial Group advised on recruitment, data acquisition, data analysis, overall methodology and/or preparation and editing of the manuscript.

Authors' information

MGW is Assistant Professor of Clinical Psychiatry (Division of Developmental Neuroscience), Pathology & Cell Biology and Pediatrics. She is a board certified child psychiatrist who has treated children with a wide range of developmental and behavioral disorders for more than 35 years with family nurture therapy. Since 1997, she has been investigating the epigenetic biologic processes and mechanisms that account for the therapeutic actions of mother-infant nurture. She is founder and co-director of the BrainGut Initiative at Columbia University Medical Center, which is translating basic research findings into testable biological treatments that will help repair or prevent childhood developmental disorders and restore physical and emotional homeostasis. The program is developing standardized strategies for clinicians and families for creating high-nurture environments to prevent or overcome childhood developmental disorders.

MMM is Chief of the Division of Developmental Neuroscience in the Department of Psychiatry at New York State Psychiatric Institute. He has conducted numerous studies in both animal models and human infant related to development of physiological systems and behavior. He is past president of the International Society of Developmental Psychobiology.

MAH is a Professor Emeritus and founder of the Division of Developmental Psychobiology (now Developmental Neuroscience) in the Department of Psychiatry at Columbia University and New York State Psychiatric Institute. He was the first Director of the Sackler Institute for Developmental Psychobiology at Columbia University, past president of the International Society of Developmental Psychobiology, and author of many basic science studies that laid the ground work for this article.

RIS is Professor of Pediatrics and Obstetrics and Gynecology. He is the past Director and Principal Investigator for the Perinatal Emphasis Research Center from the National Institute of Child Health & Development. In his animal work, Dr. Stark has studied the fetal baboon response to controlled maternal hypoxia and its effects on cardiorespiratory control. In his collaboration with the NICU Study, Dr. Stark engaged in the study design and methods. Dr. Stark brings years of clinical and translational experience to the study team, providing insights on patient care, sleep studies, and EEGs.

SAB is a research scientist at the New York State Psychiatric Institute as well as a board certified psychologist. In her animal work, Dr. Brunelli has studied the effects of mother-infant separation on behavioral and physiological development in rats. Dr. Brunelli's specialties include developmental neuroscience, child development, and child and adult neuropsychology.

HFA is an Associate Clinical Professor of Neuroscience and Biostatistics at the Mailman School of Public Health, Columbia University, and Director of the Data Coordinating Center at New York State Psychiatric Institute and Columbia University Medical Center. Dr. Andrews has extensive experience in the design of data systems and statistical analysis for clinic trials.

JA is a registered psychologist, a doctoral candidate in the Epidemiology Department, Mailman School of Public Health, Columbia University, and data analyst with the FNI-NICU study. Ms Austin has a broad experience of research, monitoring and evaluation of large international programs that concern reproductive and maternal health.

Competing interests

The authors declare that they have no competing interests.

Received: 28 July 2011 Accepted: 7 February 2012
Published: 7 February 2012

References

1. Ravn IH, Smith L, Lindemann R, Smeby NA, Kyno NM, Bunch EH, Sandvik L: **Effect of early intervention on social interaction between mothers and preterm infants at 12 months of age: A randomized controlled trial.** *Infant Behav Dev* 2011, **34(2)**:215-225.
2. Muller-Nix C, Forcada-Guex M, Pierrehumbert B, Jaunin L, Borghini A, Ansermet F: **Prematurity, maternal stress and mother-child interactions.** *Early Hum Dev* 2004, **79(2)**:145-158.
3. Meijssen D, Wolf MJ, van Bakel H, Koldewijn K, Kok J, van Baar A: **Maternal attachment representations after very preterm birth and the effect of early intervention.** *Infant Behav Dev* 2011, **34(1)**:72-80.
4. Meijssen D, Wolf MJ, Koldewijn K, Houtzager BA, van Wassenaer A, Tronick E, Kok J, van Baar A: **The effect of the Infant Behavioral Assessment and Intervention Program on mother-infant interaction after very preterm birth.** *J Child Psychol Psychiatry* 2010, **51(11)**:1287-1295.
5. Shah PE, Clements M, Poehlmann J: **Maternal resolution of grief after preterm birth: implications for infant attachment security.** *Pediatrics* 2011, **127(2)**:284-292.
6. Coppola G, Cassibba R, Costantini A: **What can make the difference? Premature birth and maternal sensitivity at 3 months of age: the role of attachment organization, traumatic reaction and baby's medical risk.** *Infant Behav Dev* 2007, **30(4)**:679-684.
7. Hofer MA: **Early relationships as regulators of infant physiology and behavior.** *Acta Paediatr Suppl* 1994, **397**:9-18.

8. Hofer MA: **Hidden regulators in attachment, separation, and loss.** *Monogr Soc Res Child Dev* 1994, **59(2-3)**:192-207.
9. Hane AA, Fox NA: **Ordinary variations in maternal caregiving influence human infants' stress reactivity.** *Psychol Sci* 2006, **17(6)**:550-556.
10. Hane AA, Henderson HA, Reeb-Sutherland BC, Fox NA: **Ordinary variations in human maternal caregiving in infancy and biobehavioral development in early childhood: A follow-up study.** *Dev psychobiol* 2010, **52(6)**:558-567.
11. Schore AN: **Back to basics: attachment, affect regulation, and the developing right brain: linking developmental neuroscience to pediatrics.** *Pediatr Rev* 2005, **26(6)**:204-217.
12. Moore GA, Hill-Soderlund AL, Propper CB, Calkins SD, Mills-Koonce WR, Cox MJ: **Mother-infant vagal regulation in the face-to-face still-face paradigm is moderated by maternal sensitivity.** *Child Dev* 2009, **80(1)**:209-223.
13. Haley DW, Stansbury K: **Infant stress and parent responsiveness: regulation of physiology and behavior during still-face and reunion.** *Child Dev* 2003, **74(5)**:1534-1546.
14. Forcada-Guex M, Pierrehumbert B, Borghini A, Moessinger A, Muller-Nix C: **Early dyadic patterns of mother-infant interactions and outcomes of prematurity at 18 months.** *Pediatrics* 2006, **118(1)**:e107-114.
15. Bystrova K, Ivanova V, Edhborg M, Matthiesen AS, Ransjo-Arvidson AB, Mukhamedrakhimov R, Uvnas-Moberg K, Widstrom AM: **Early contact versus separation: effects on mother-infant interaction one year later.** *Birth* 2009, **36(2)**:97-109.
16. Beckwith L, Rodning C: **Dyadic processes between mothers and preterm infants: Development at ages 2 to 5 years.** *Inf Mental Hlth J* 1996, **17(4)**:322-333.
17. Brown L: **Heart rate variability in premature infants during feeding.** *Biol Res Nurs* 2007, **8(4)**:283-293.
18. Welch MG, Northrup RS, Welch-Horan TB, Ludwig RJ, Austin CL, Jacobson JS: **Outcomes of Prolonged Parent-Child Embrace Therapy among 102 children with behavioral disorders.** *Complement Ther Clin Pract* 2006, **12(1)**:3-12.
19. Welch MG, Chaput P: **Mother-child holding therapy and autism.** *Pa Med* 1988, **91(10)**:33-38.
20. Welch MG: **Holding Time: How to Eliminate Conflict, Temper Tantrums, and Sibling Rivalry and Raise Happy, Loving, Successful children.** New York, NY: Simon and Schuster; 1988.
21. Welch MG: **Toward prevention of developmental disorders.** *Pa Med* 1987, **90(3)**:47-52.
22. Feldman R, Eidelman AI: **Skin-to-skin contact (Kangaroo Care) accelerates autonomic and neurobehavioural maturation in preterm infants.** *Dev Med Child Neurol* 2003, **45(4)**:274-281.
23. Feldman R, Eidelman AI, Sirota L, Weller A: **Comparison of skin-to-skin (kangaroo) and traditional care: parenting outcomes and preterm infant development.** *Pediatrics* 2002, **110(1 Pt 1)**:16-26.
24. Feldman R, Weller A, Sirota L, Eidelman AI: **Skin-to-Skin contact (Kangaroo care) promotes self-regulation in premature infants: sleep-wake cyclicity, arousal modulation, and sustained exploration.** *Dev Psychol* 2002, **38(2)**:194-207.
25. Melnyk BM, Feinstein NF: **Reducing hospital expenditures with the COPE (Creating Opportunities for Parent Empowerment) program for parents and premature infants: an analysis of direct healthcare neonatal intensive care unit costs and savings.** *Nurs Adm Q* 2009, **33(1)**:32-37.
26. Melnyk BM, Feinstein NF, Alpert-Gillis L, Fairbanks E, Crean HF, Sinkin RA, Stone PW, Small L, Tu X, Gross SJ: **Reducing premature infants' length of stay and improving parents' mental health outcomes with the Creating Opportunities for Parent Empowerment (COPE) neonatal intensive care unit program: a randomized, controlled trial.** *Pediatrics* 2006, **118(5)**:e1414-1427.
27. Dunn MS, Reilly MC, Johnston AM, Hoopes RD Jr, Abraham MR: **Development and dissemination of potentially better practices for the provision of family-centered care in neonatology: the family-centered care map.** *Pediatrics* 2006, **118(Suppl 2)**:S95-107.
28. Johnston AM, Bullock CE, Graham JE, Reilly MC, Rocha C, Hoopes RD Jr, Van der Meid V, Gutierrez S, Abraham MR: **Implementation and case-study results of potentially better practices for family-centered care: the family-centered care map.** *Pediatrics* 2006, **118(Suppl 2)**:S108-114.
29. Vanderveen JA, Bassler D, Robertson CM, Kirpalani H: **Early interventions involving parents to improve neurodevelopmental outcomes of premature infants: a meta-analysis.** *J Perinatol* 2009, **29(5)**:343-351.
30. Porter RH: **Olfaction and human kin recognition.** *Genetica* 1998, **104(3)**:259-263.
31. Porter RH, Winberg J: **Unique salience of maternal breast odors for newborn infants.** *Neurosci Biobehav Rev* 1999, **23(3)**:439-449.
32. Schaal B: **Mammary odor cues and pheromones: mammalian infant-directed communication about maternal state, mammae, and milk.** *Vitam Horm* 2010, **83**:83-136.
33. Cernoch JM, Porter RH: **Recognition of maternal axillary odors by infants.** *Child Dev* 1985, **56(6)**:1593-1598.
34. Nishitani S, Miyamura T, Tagawa M, Sumi M, Takase R, Doi H, Moriuchi H, Shinohara K: **The calming effect of a maternal breast milk odor on the human newborn infant.** *Neurosci Res* 2009, **63(1)**:66-71.
35. Rattaz C, Goubet N, Bullinger A: **The calming effect of a familiar odor on full-term newborns.** *J Dev Behav Pediatr* 2005, **26(2)**:86-92.
36. Sullivan RM, Toubas P: **Clinical usefulness of maternal odor in newborns: soothing and feeding preparatory responses.** *Biol Neonate* 1998, **74(6)**:402-408.
37. Porter RH, Cernoch JM, Perry S: **The importance of odors in mother-infant interactions.** *Matern Child Nurs J* 1983, **12(3)**:147-154.
38. Mills M, Melhuish E: **Recognition of mother's voice in early infancy.** *Nature* 1974, **252(5479)**:123-124.
39. DeCasper AJ, Fifer WP: **Of human bonding: newborns prefer their mothers' voices.** *Science* 1980, **208(4448)**:1174-1176.
40. Ockleford EM, Vince MA, Layton C, Reader MR: **Responses of neonates to parents' and others' voices.** *Early Hum Dev* 1988, **18(1)**:27-36.
41. Therien JM, Worwa CT, Mattia FR, deRegnier RA: **Altered pathways for auditory discrimination and recognition memory in preterm infants.** *Dev Med Child Neurol* 2004, **46(12)**:816-824.
42. Livingston K, Beider S, Kant AJ, Gallardo CC, Joseph MH, Gold JI: **Touch and massage for medically fragile infants.** *Evid based complement alternat med: eCAM* 2009, **6(4)**:473-482.
43. Guzzetta A, Baldini S, Bancale A, Baroncelli L, Ciucci F, Ghirri P, Putignano E, Sale A, Viegi A, Berardi N, *et al*: **Massage accelerates brain development and the maturation of visual function.** *J Neurosci* 2009, **29(18)**:6042-6051.
44. Diego MA, Field T, Hernandez-Reif M: **Procedural pain heart rate responses in massaged preterm infants.** *Infant Behav Dev* 2009, **32(2)**:226-229.
45. Diego MA, Field T, Hernandez-Reif M, Deeds O, Ascencio A, Begert G: **Preterm infant massage elicits consistent increases in vagal activity and gastric motility that are associated with greater weight gain.** *Acta Paediatr* 2007, **96(11)**:1588-1591.
46. Gonzalez AP, Vasquez-Mendoza G, Garcia-Vela A, Guzman-Ramirez A, Salazar-Torres M, Romero-Gutierrez G: **Weight gain in preterm infants following parent-administered Vimala massage: a randomized controlled trial.** *Am J Perinatol* 2009, **26(4)**:247-252.
47. Nagy E, Molnar P: **Heart rate deceleration during the grasping reflex.** *Eur J Pediatr* 1999, **158(7)**:576-577.
48. Nyqvist KH, Anderson GC, Bergman N, Cattaneo A, Charpak N, Davanzo R, Ewald U, Ludington-Hoe S, Mendoza S, Pallas-Allonso C, *et al*: **State of the art and recommendations. Kangaroo mother care: application in a high-tech environment.** *Acta paediatrica* 2010, **99(6)**:812-819.
49. Kim KH, Relkin NR, Lee KM, Hirsch J: **Distinct cortical areas associated with native and second languages.** *Nature* 1997, **388(6638)**:171-174.
50. McAnulty G, Duffy FH, Butler S, Parad R, Ringer S, Zurakowski D, Als H: **Individualized developmental care for a large sample of very preterm infants: health, neurobehaviour and neurophysiology.** *Acta paediatrica* 2009, **98(12)**:1920-1926.
51. McDonald SD, Han Z, Mulla S, Ohlsson A, Beyene J, Murphy KE: **Preterm birth and low birth weight among in vitro fertilization twins: a systematic review and meta-analyses.** *Eur J Obstet Gynecol Reprod Biol* 2010, **148(2)**:105-113.
52. Beebe B, Jaffe J, Markese S, Buck K, Chen H, Cohen P, Bahrick L, Andrews H, Feldstein S: **The origins of 12-month attachment: a microanalysis of 4-month mother-infant interaction.** *Attach Hum Dev* 2010, **12(1-2)**:3-141.
53. Spielberger CD, Gorsuch RL, Lushene RE: **STAI manual for the State-Trait Anxiety Inventory ("self-evaluation questionnaire").** Palo Alto, CA: Consulting Psychologists Press; 1970.

54. Radloff LS: **The CES-D Scale: A Self-Report Depression Scale for Research in the General Population.** *Appl Psych Meas* 1977, **1**:385-401.
55. George C, Kaplan N, Main M: **Adult Attachment Interview.** Berkeley, CA: University of California, Berkeley; 1985.
56. Carver CSW: **Behavioral inhibition, behavioral activation, and affective responses to impending reward and punishment: The BIS/BAS scales.** *Journal of Personality and Social Psychology* 1994, **67**:319-333.
57. Abidin RR: **Parenting Stress Index.** Charlottesville, VA: Pediatric Psychology Press; 1990.
58. Loyd BH, Abidin RR: **Revision of the Parenting Stress Index.** *J Pediatr Psychol* 1985, **10(2)**:169-177.
59. Norbeck JS, Lindsey AM, Carrieri VL: **Further development of the Norbeck Social Support Questionnaire: normative data and validity testing.** *Nurs Res* 1983, **32(1)**:4-9.
60. Teti DM, Gelfand DM: **Behavioral Competence among Mothers of Infants in the 1st Year-the Mediational Role of Maternal Self-Efficacy.** *Child Dev* 1991, **62(5)**:918-929.
61. Derryberry D, Rothbart MK: **Arousal, affect, and attention as components of temperament.** *J Pers Soc Psychol* 1988, **55(6)**:958-966.
62. Tronick EZ, Lester BM: **The NICU Network Neurobehavioral Scale: a comprehensive instrument to assess substance-exposed and high-risk infants.** *NIDA Res Monogr* 1996, **166**:198-204.
63. Lester BM, Tronick EZ, Brazelton TB: **The Neonatal Intensive Care Unit Network Neurobehavioral Scale procedures.** *Pediatrics* 2004, **113(3 Pt 2)**:641-667.
64. Medoff-Cooper B, Carey WB, McDevitt SC: **The Early Infancy Temperament Questionnaire.** *J Dev Behav Pediatr* 1993, **14(4)**:230-235.
65. Parade SH, Leerkes EM: **The reliability and validity of the Infant Behavior Questionnaire-Revised.** *Infant Behav Dev* 2008, **31(4)**:637-646.
66. Briggs-Gowan MJ, Carter AS, Irwin JR, Wachtel K, Cicchetti DV: **The Brief Infant-Toddler Social and Emotional Assessment: screening for social-emotional problems and delays in competence.** *J Pediatr Psychol* 2004, **29(2)**:143-155.
67. Achenbach TM: **Manual for the child behavior checklist/2-3 and 1992 profile.** Burlington, VT: Department of Psychiatry, University of Vermont; 1992.
68. Squires J, Bricker D, Potter L: **Revision of a parent-completed development screening tool: Ages and Stages Questionnaires.** *J Pediatr Psychol* 1997, **22(3)**:313-328.
69. Bayley N: **Bayley Scales of Infant and Toddler Development.** San Antonio, TX: Harcourt Assessment; 2006.
70. Apgar V: **A proposal for a new method of evaluation of the newborn infant.** *Curr Res Anesth Analg* 1953, **32(4)**:260-267.
71. Stefanski M, Schulze K, Bateman D, Kairam R, Pedley TA, Masterson J, James LS: **A scoring system for states of sleep and wakefulness in term and preterm infants.** *Pediatr Res* 1984, **18(1)**:58-62.
72. Grieve PG, Emerson RG, Fifer WP, Isler JR, Stark RI: **Spatial correlation of the infant and adult electroencephalogram.** *Clin Neurophysiol* 2003, **114(9)**:1594-1608.
73. Grieve PG, Isler JR, Izraelit A, Peterson BS, Fifer WP, Myers MM, Stark RI: **EEG functional connectivity in term age extremely low birth weight infants.** *Clin Neurophysiol* 2008, **119(12)**:2712-2720.
74. Bettendorf M, Albers N, Bauer J, Heinrich UE, Linderkamp O, Maser-Gluth C: **Longitudinal evaluation of salivary cortisol levels in full-term and preterm neonates.** *Horm Res* 1998, **50(6)**:303-308.
75. Morelius E, Hellstrom-Westas L, Carlen C, Norman E, Nelson N: **Is a nappy change stressful to neonates?** *Early Hum Dev* 2006, **82(10)**:669-676.
76. Ainsworth MDS: **Technical manual for the systems for coding infant attachment and reciprocal maternal behaviors.** Princeton, NJ: Educational Testing Service; 1976.
77. Tronick E, Als H, Adamson L, Wise S, Brazelton TB: **The infant's response to entrapment between contradictory messages in face-to-face interaction.** *J Am Acad Child Psychiatry* 1978, **17(1)**:1-13.
78. Zimmerman IL, Steiner VG, Pond RE: **Preschool Language Scale, 5th edition.** San Antonio, TX: The Psychological Corporation; 2011.
79. Napiorkowski B, Lester BM, Freier MC, Brunner S, Dietz L, Nadra A, Oh W: **Effects of in utero substance exposure on infant neurobehavior.** *Pediatrics* 1996, **98(1)**:71-75.
80. Lester BM, Tronick EZ: **History and description of the Neonatal Intensive Care Unit Network Neurobehavioral Scale.** *Pediatrics* 2004, **113(3 Pt 2)**:634-640.
81. Ross CE, Mirowsky J: **Components of depressed mood in married men and women. The Center for Epidemiologic Studies' Depression Scale.** *Am J Epidemiol* 1984, **119(6)**:997-1004.
82. Hofer MA: **Psychobiological Roots of Early Attachment.** *Curr Dir Psychol Sci* 2006, **15**:84-88.
83. Brunelli SA: **Development and evolution of hidden regulators: selective breeding for an infantile phenotype.** *Dev psychobiol* 2005, **47(3)**:243-252.
84. Hofer MA: **On the nature and consequences of early loss.** *Psychosom Med* 1996, **58(6)**:570-581.
85. Meaney MJ: **Maternal care, gene expression, and the transmission of individual differences in stress reactivity across generations.** *Annu Rev Neurosci* 2001, **24**:1161-1192.
86. Sanchez MM, Ladd CO, Plotsky PM: **Early adverse experience as a developmental risk factor for later psychopathology: evidence from rodent and primate models.** *Dev Psychopathol* 2001, **13(3)**:419-449.
87. Higgins JPT, Green S: **Cochrane Handbook for Systematic Reviews of Interventions.** 2009, vol. Version 5.0.2 (updated September 2009): The Cochrane Collaboration.
88. Tronick EZ, Olson K, Rosenberg R, Bohne L, Lu J, Lester BM: **Normative neurobehavioral performance of healthy infants on the Neonatal Intensive Care Unit Network Neurobehavioral Scale.** *Pediatrics* 2004, **113(3 Pt 2)**:676-678.
89. Morelius E, Nelson N, Gustafsson PA: **Salivary cortisol response in mother-infant dyads at high psychosocial risk.** *Child Care Health Dev* 2007, **33(2)**:128-136.
90. Crawford JR, Cayley C, Wilson PH, Lovibond PF, Hartley C: **Percentile norms and accompanying interval estimates from an Australian general adult population sample for self report mood scales (BAI, BDI, CRSD, CES-D, DASS, DASS-21, STAI-X, STAI-Y, SRDS, and SRAS).** *The Australian Psychologist* 2011, **46**:3-14.
91. Miles MS, Holditch-Davis D, Schwartz TA, Scher M: **Depressive symptoms in mothers of prematurely born infants.** *J dev behav pediatr: JDBP* 2007, **28(1)**:36-44.
92. Sahni R, Schulze KF, Kashyap S, Ohira-Kist K, Fifer WP, Myers MM: **Maturational changes in heart rate and heart rate variability in low birth weight infants.** *Dev Psychobiol* 2000, **37(2)**:73-81.
93. Sahni R, Schulze KF: **Sleep state, cardiorespiratory and electrocortical activity in infants with transposition of great vessels.** *Neuropediatrics* 1997, **28(3)**:162-167.
94. Sahni R, Schulze KF, Ohira-Kist K, Kashyap S, Myers MM, Fifer WP: **Interactions among peripheral perfusion, cardiac activity, oxygen saturation, thermal profile and body position in growing low birth weight infants.** *Acta paediatrica* 2010, **99(1)**:135-139.
95. Neu M, Laudenslager ML, Robinson J: **Coregulation in salivary cortisol during maternal holding of premature infants.** *Biol Res Nurs* 2009, **10(3)**:226-240.
96. Palmer C, Bik EM, DiGiulio DB, Relman DA, Brown PO: **Development of the human infant intestinal microbiota.** *PLoS Biol* 2007, **5(7)**:e177.

Pre-publication history
The pre-publication history for this paper can be accessed here:
http://www.biomedcentral.com/1471-2431/12/14/prepub

doi:10.1186/1471-2431-12-14
Cite this article as: Welch *et al.*: Family nurture intervention (FNI): methods and treatment protocol of a randomized controlled trial in the NICU. *BMC Pediatrics* 2012 **12**:14.
